# Neutrophil extracellular traps promote neuronal ferroptosis through STING-mediated AMPK dysregulation after traumatic brain injury

**DOI:** 10.7150/ijbs.130882

**Published:** 2026-07-30

**Authors:** Guihong Shi, Jianye Xu, Yue Tian, Yiyao Cao, Shaochen Yu, Qifeng Li, Luyuan Zhang, Kaiyuan Huang, Rui Tan, Xiujue Zheng, Renya Zhan

**Affiliations:** 1 Department of Neurosurgery, The First Affiliated Hospital, College of Medicine, Zhejiang University, Hangzhou 310003, Zhejiang, China.; 2 Department of Neurosurgery, Qilu Hospital, Cheeloo College of Medicine and Institute of Brain and Brain-Inspired Science, Shandong University, Jinan, China.; 3 Department of Neurosurgery, Tianjin Medical University General Hospital, Tianjin 300052, China.; 4 Department of Neurosurgery, Provincial Hospital Affiliated to Shandong First Medical University, Jinan, Shandong 250021, China.

**Keywords:** traumatic brain injury, neutrophil extracellular traps, ferroptosis, STING, AMPK, lipid peroxidation

## Abstract

Neuronal ferroptosis exacerbates neurological dysfunction after traumatic brain injury (TBI), yet the upstream signals driving this process remain poorly defined. Here, we identify neutrophil extracellular traps (NETs) as critical mediators of post-TBI ferroptotic injury. In patients, NET accumulation closely accompanied markers of neuronal ferroptosis and correlated with neurological severity. In a mouse TBI model, genetic ablation of peptidylarginine deiminase 4 (PAD4) or enzymatic degradation of NET-derived DNA markedly attenuated neuronal lipid peroxidation, suppressed ferroptosis-associated neuronal injury, and improved functional outcomes. Mechanistically, NET-derived DNA activated neuronal stimulator of interferon genes (STING) signaling, leading to suppression of intrinsic antioxidant defenses and amplification of lipid peroxidation. Pharmacological inhibition of STING or activation of AMP-activated protein kinase (AMPK) signaling effectively counteracted NET-induced ferroptotic stress and preserved neuronal viability. Collectively, these findings establish NET-derived DNA as a key upstream regulator of neuronal ferroptosis after TBI and highlight neutrophil-driven oxidative stress as a promising therapeutic target for secondary brain injury.

## Introduction

Traumatic brain injury (TBI) is a major cause of disability and long-term neurological impairment worldwide^1^, ^2^. Its pathological progression involves an initial mechanical insult, followed by a prolonged and complex cascade of secondary brain injury^3^, ^4^. The primary injury refers to the instantaneous physical disruption of brain tissue and acute neuronal necrosis. The secondary brain injury evolves over hours to weeks and is characterized by the progressive deterioration of the peri-lesional microenvironment and brain tissue damage. Robust neuroinflammatory activation, oxidative and metabolic stress, hypoxia-reoxygenation injury, and the accumulation of cytotoxic molecules collectively exacerbate tissue damage^5^-^7^. These pathological events ultimately lead to further neuronal loss through apoptosis and multiple regulated cell death pathways, including necroptosis, pyroptosis, ferroptosis, and autophagy-dependent cell death^8^-^10^. Secondary neuronal death has been shown to largely determine long-term functional outcomes, making it critical to elucidate the upstream drivers and molecular mechanisms that exacerbate neuronal vulnerability for the development of effective therapeutic strategies.

Although inhibition of apoptotic, necroptotic, and pyroptotic cell death has been shown to improve outcomes in experimental models of TBI, effective strategies targeting these pathways have not yet been successfully translated into clinical use. Ferroptosis is an iron-dependent form of regulated cell death characterized by lipid peroxidation and catastrophic membrane damage, and has recently been identified as an important contributor to neuronal loss after TBI^11^-^13^. Mechanistically, ferroptosis differs from apoptosis and necroptosis because it is primarily driven by iron-dependent lipid peroxidation and impaired antioxidant defense rather than caspase-dependent execution or RIPK1/RIPK3/MLKL-mediated necrotic signaling. Neurons are particularly susceptible to ferroptosis due to their high oxygen consumption, membrane enrichment in polyunsaturated fatty acids, and limited antioxidant capacity^14^, ^15^. Following TBI, dysregulated iron homeostasis, glutathione depletion, mitochondrial dysfunction, and excessive reactive oxygen species production further exacerbate ferroptosis-associated injury^9^, ^16^, ^17^. Evidence from both animal and clinical studies indicates that ferroptosis contributes to lesion expansion, aggravated neuroinflammation, and impaired functional recovery^18^, ^19^. In experimental TBI models, pharmacological inhibition of ferroptosis has been shown to mitigate oxidative damage and improve neurological outcomes, highlighting ferroptosis as a critical pathological process^20^. Therefore, ferroptosis was selected as the focus of this study because it represents a mechanistically distinct and therapeutically relevant form of neuronal injury in the oxidatively stressed post-TBI brain. However, the upstream inflammatory triggers that sensitize neurons to ferroptosis remain incompletely understood.

Neutrophils rapidly infiltrate the injured brain and amplify tissue damage through the release of proteases, reactive oxygen species (ROS), and proinflammatory mediators^21^, ^22^. A major effector mechanism is the formation of neutrophil extracellular traps (NETs), which consist of chromatin fibers decorated with histones and granular enzymes^23^, ^24^. Beyond extracellular DNA, NET-associated proteins may also contribute to neural injury. For example, extracellular histones can exert direct cytotoxic and barrier-disruptive effects, whereas myeloperoxidase and neutrophil elastase may amplify oxidative stress, proteolytic tissue damage, and neuroinflammatory responses. NETs have recently been implicated as key drivers of sterile neuroinflammation^25^-^29^. Clinical and experimental evidence demonstrates marked NET accumulation after TBI and links NETs to coagulopathy, neuroinflammation, blood-brain barrier (BBB) breakdown, and neuronal injury^24^, ^30^-^32^. Our previous work also identified acute NET formation as a critical contributor to secondary brain injury and neurological deficits^5^, ^33^, ^34^. A major bioactive component of NETs, extracellular double-stranded DNA, acts as a potent damage-associated signal that activates the cyclic GMP-AMP synthase-stimulator of interferon genes (cGAS-STING) pathway^33^. STING signaling promotes mitochondrial stress, ROS generation, iron dysregulation, and lipid peroxidation, and has been implicated in neuronal ferroptosis across central nervous system (CNS) injury models^35^, ^36^. However, whether NET-derived extracellular double-stranded DNA (dsDNA) activates STING after TBI to drive neuronal ferroptosis via this NET-STING axis remains unknown.

In this study, we define the upstream role of NET-derived dsDNA in triggering STING activation and promoting neuronal ferroptosis after TBI. We demonstrate that dsDNA-STING signaling amplifies ROS production and lipid peroxidation through the AMPK-Nrf2-HO-1 pathway, thereby heightening neuronal susceptibility to ferroptosis-associated neuronal injury. These findings identify a mechanistic link between neutrophil activation and neuronal damage, revealing the NET-STING axis as a promising therapeutic target for mitigating secondary brain injury.

## Methods

### Human brain tissue and blood samples

Human brain tissues were collected at Tianjin Medical University General Hospital, China, between June 2021 and June 2025 under an ethically approved protocol, with written informed consent obtained from all participants or their legally authorized representatives. Eight severe TBI samples (GCS < 9) were obtained during decompressive craniotomy for hematoma evacuation and/or management of life-threatening intracranial hypertension, and four non-contusive control samples were collected from patients undergoing cerebrovascular malformation surgery. Demographic and clinical characteristics are summarized in **Supplementary Table S1.**

Peripheral blood was collected from 24 severe TBI patients and 12 healthy volunteers within 24 h post-injury, processed to remove platelets, and plasma stored at -80 °C. A subset of samples (7 patients, 7 controls) was used for neutrophil isolation for *in vitro* experiments. Exclusion criteria included hepatic or renal insufficiency, hematologic disorders, active infections, pregnancy, or prior anticoagulant/antiplatelet therapy. All specimens were de-identified and coded to ensure confidentiality. The study was conducted in accordance with the Declaration of Helsinki and approved by the Ethics Committee of Tianjin Medical University.

### Human neutrophil isolation and *in vitro* NET assay

Peripheral blood samples were obtained from patients with TBI and healthy donors. Human neutrophils were isolated using magnetic-activated cell sorting (MACS) according to the manufacturer's instructions. Briefly, peripheral blood cells were incubated with a Neutrophil Isolation Kit (Miltenyi Biotec, Germany), followed by magnetic separation using a MACS Separator to obtain purified neutrophils. After isolation, neutrophils were washed and resuspended in RPMI-1640 medium (Gibco, USA). Purified neutrophils were seeded onto glass-bottom culture plates at a density of 5 × 10⁵ cells/mL and allowed to rest for 30 min at 37 °C in a humidified incubator with 5% CO₂. Cells were then stimulated with lipopolysaccharide (LPS, 10 μg/mL; Sigma-Aldrich, USA) for 4 h to induce NET formation. Control cells were treated with PBS. Following stimulation, neutrophils were fixed with 2% paraformaldehyde and subjected to immunofluorescence staining. After blocking with 3% BSA in PBS, cells were incubated with primary antibodies against H3cit overnight at 4 °C, followed by appropriate fluorescent secondary antibodies. Nuclear DNA was counterstained with DAPI. NET formation was evaluated by fluorescence microscopy. In parallel experiments, freshly isolated neutrophils were lysed immediately for western blot analysis as indicated.

### Animals

Adult male C57BL/6J mice (8 weeks old, 20-25 g) were purchased from the Experimental Animal Laboratories of the Academy of Military Medical Sciences (Beijing, China). PAD4-/- mice on a C57BL/6J background were obtained from The Jackson Laboratory, with age- and sex-matched wild-type littermates used as controls. Mice were housed in specific-pathogen-free conditions with controlled temperature (20-24 °C) and humidity (50-60%), maintained on a 12 h light/dark cycle, and fed with a standard diet ad libitum for at least one week prior to experimentation to allow acclimation and minimize stress. All experimental procedures involving animals were approved by the Animal Care and Use Committee of Tianjin Medical University and performed in accordance with the National Institutes of Health Guide for the Care and Use of Laboratory Animals.

### Controlled cortical impact (CCI) model of traumatic brain injury

TBI was induced using a digitally controlled cortical impact (CCI) system (eCCI-6.3, Custom Design & Fabrication, USA), following previously established protocols. Mice were anesthetized with 3% isoflurane in a 7:3 mixture of nitrous oxide and oxygen for induction, and anesthesia was maintained with 1.5% isoflurane via a nose cone. Body temperature was maintained at 37 ± 0.5 °C using a heating pad. Animals were secured in a stereotaxic frame in the prone position. A 3.5-mm diameter craniotomy was performed over the right parietotemporal region (2.5 mm posterior to the bregma and 2.5 mm lateral to the sagittal suture) using a motorized drill, following a midline scalp incision to expose the skull. A vertically directed CCI was applied using a 4-mm flat-tip impactor (5 m/s velocity, 200 ms dwell time, 2.2 mm depth). After the injury, the skin incision was closed, and mice were placed on a heating pad to recover from anesthesia. Sham-operated mice underwent the identical craniotomy procedure without cortical impact.

### Randomization and blinding

For all *in vivo* experiments, mice were randomly assigned to experimental groups before surgery or pharmacological intervention. Drug solutions, vehicle controls, and viral vectors were prepared and coded by an investigator who was not involved in outcome assessment. Investigators performing neurobehavioral testing, tissue processing, histological and immunofluorescence quantification, biochemical assays, western blot densitometry, and statistical analyses were blinded to group allocation. Group identities were decoded only after data acquisition and primary analyses were completed. Because sham and CCI procedures are technically distinguishable during model induction, the surgeon could not be fully blinded to injury status; however, all subsequent outcome assessments and analyses were performed in a blinded manner.

### Pharmacological modulation of STING and AMPK-Nrf2 signaling *in vivo*

To modulate STING and AMPK-Nrf2 signaling *in vivo*, pharmacological agents were administered according to prior studies. Dorsomorphin (5 μg/mouse, S7306, Selleck, USA), an AMPK inhibitor, was dissolved in DMSO and administered intravenously (i.v.) once daily for three consecutive days, beginning 30 min before CCI^5^. To activate AMPK signaling, mice received the AMPK agonist A-769662 (25 mg/kg; Selleck, USA) by intraperitoneal injection beginning 30 min after TBI and then once daily for three consecutive days^37^. As previously described, mice were intravenously injected with recombinant human DNase I (5 mg/kg; ProSpec, Israel) 6 h after TBI and subsequently once daily until sacrifice^5^. For STING pathway modulation, 2′3′-cGAMP (1 mg/kg; InvivoGen, USA) was administered intravenously 10 min before TBI and again at 24 h and 48 h after injury^33^. This pre-injury administration was designed as a mechanistic gain-of-function approach rather than a clinically intended therapeutic regimen. Because STING activation occurs early during the acute secondary injury cascade after TBI, 2′3′-cGAMP was administered 10 min before CCI to ensure sufficient STING pathway activation at the onset of injury and to test whether forced STING activation could reverse the protection conferred by PAD4 deficiency. Repeated injections at 24 h and 48 h were used to maintain STING activation during the acute post-injury phase. The STING antagonist C-176 (10 mg/kg; Selleck, USA) was dissolved in 5% DMSO and 95% corn oil, diluted in saline (final 5% v/v), and administered intraperitoneally (i.p.) 1 h after TBI and then once daily until sacrifice^33^. To inhibit Nrf2 signaling, ML385 (30 mg/kg; Selleck, USA) was administered i.p. using the same dosing schedule^38^.

### Injection of adenoviruses

Recombinant PAD4 adenovirus (Ad-PAD4; Adeno-CMV-MCS-Padi4-3×FLAG-SV40-EGFP, serotype 5, 4 × 10^10^ PFU/mL) and control adenovirus (Ad-con) were purchased from Genechem(Shanghai, China). Based on previous studies showing that intracerebral adenoviral transgene expression is initiated within the first day and reaches relatively high levels approximately 2-4 days after injection^39^, and because our analyses focused on the acute post-TBI phase, particularly 3 days after CCI, adenoviruses were injected 24 h before CCI. This schedule allowed PAD4 expression to be initiated by the time of injury and to remain elevated during the early post-traumatic window, coinciding with the period of robust neutrophil infiltration, NET formation, STING activation, and ferroptosis-associated neuronal injury. Stereotactic intracortical injection was performed as described in our previous study with minor modifications^5^, ^33^. Briefly, a total of 4 μL of Ad-PAD4 or Ad-con was injected into the right cortex at two sites (1.5 mm rostral and 1.5 mm caudal to the planned impact location; depth 1.5 mm) using a 5-μL Hamilton syringe at a rate of 0.5 μL/min. The needle was kept in place for 10-15 min to prevent reflux before withdrawal. All injections were performed by investigators blinded to group allocation.

### Neurobehavioral assessment

To evaluate short- and long-term functional outcomes, neurobehavioral assessments were conducted in all groups by investigators blinded to group allocation. Four standardized tests were administered systematically according to the following protocol:

**The modified neurological severity score (mNSS)** was used to quantify neurological deficits according to a previously established 18-point scoring protocol^33^. This composite scale evaluates motor function, sensory responses, reflexes/abnormal movements, and beam-balance performance. Motor function contributed up to 6 points, sensory responses up to 2 points, reflexes and abnormal movements up to 4 points, and beam-balance performance up to 6 points. One point was assigned for each failed task, abnormal behavior, or absent reflex, and the final mNSS was calculated as the sum of all components. An overall score of 0 indicated normal neurological function, whereas scores of 1-6, 7-12, and 13-18 represented mild, moderate, and severe neurological deficits, respectively. Evaluations were performed at days 1, 3, 5, 7, 14 and 21 post-CCI, and preoperative baseline measurements were obtained prior to CCI induction.

**Rotarod test** was performed to evaluate motor coordination and balance using an accelerating Rotarod apparatus (RWD Life Science, Shenzhen, China), as described previously^33^. Before CCI induction, mice underwent pre-injury acclimation and training for 3 consecutive days. On each training day, mice completed three trials on the rotating rod, with an inter-trial interval of at least 30 min to prevent fatigue. Each trial consisted of acceleration from 4 to 40 rpm over 300 s. Stable baseline performance was considered achieved when the latency to fall showed no obvious fluctuation across the final training trials, and the mean latency from the last training session was used as the preoperative baseline. At each post-injury testing time point, mice completed three consecutive trials on a rod accelerating linearly from 4 to 40 rpm over 300 s, with 5-min inter-trial intervals. Latency to fall was automatically recorded using infrared sensors, and the mean of triplicate measurements was used for analysis.

**Cylinder test** was employed to assess forelimb motor asymmetry after CCI. The test was performed preoperatively to establish a baseline and then repeated on post-CCI days 1, 3, 5, 7, 14 and 21. Mice were acclimated to the testing environment for at least 30 min before being placed individually into a transparent acrylic cylinder (9 cm diameter × 38 cm height). Vertical exploratory behavior was recorded from above for 10 min. The number of left, right, and simultaneous forelimb wall contacts during rearing was counted from video, and forelimb asymmetry was calculated using the formula: (right - left) / (left + right + both) × 100%, as described previously^33^.

**Foot fault test** was used to evaluate the sensorimotor function of the forepaw and hindpaw, as described previously^40^. During the test, mice were allowed to freely walk on a metal grid for 3 min, and a foot fault was recorded when the contralateral forepaw or hindpaw slipped or fell between the grid openings. The test was performed 1 day before surgery and repeated on post-CCI days 1, 3, 5, 7, 14, and 21. Functional performance was quantified as the percentage of error steps of the contralateral forepaw or hindpaw relative to the corresponding total number of steps.

**Morris water maze (MWM) test** was conducted to assess long-term spatial learning and memory on days 15-21 post-CCI. The circular pool (120 cm diameter, 50 cm depth) was filled with opaque water (22 ± 1 °C) and surrounded by four distal visual cues. During the acquisition phase (days 15-20), mice underwent four daily trials initiated from pseudo-randomized starting locations to locate a submerged escape platform (10 cm diameter) positioned 1 cm below the water surface. Each trial lasted up to 90 s, and mice failing to locate the platform were guided to it for a 15-s orientation period. On day 21, a probe trial was conducted with the platform removed, and mice were allowed to swim freely for 90 s from a starting point opposite the former platform quadrant. Swimming trajectories, escape latency, platform crossings, target-quadrant dwell time, and swim speed were recorded and analyzed using a video tracking system (EthoVision XT 13, Noldus Information Technology, Wageningen, the Netherlands).

### Enzyme-linked immunosorbent assay (ELISA) and biochemical assays

Levels of NET-associated markers, neuronal injury-related proteins, and ferroptosis-related parameters were measured using commercially available enzyme-linked immunosorbent assay (ELISA) kits or biochemical assay kits according to the manufacturers' instructions.

For human samples, peripheral blood was collected from patients with traumatic brain injury and healthy controls, and plasma was isolated by centrifugation and stored at -80 °C until analysis. Plasma levels of cell-free DNA, MPO-DNA complexes, H3cit, UCHL1, NEFL, as well as Fe²⁺ and malondialdehyde (MDA) were determined. For animal experiments, mouse blood samples were collected at the indicated time points, and plasma was prepared by centrifugation at 500 × g for 20 min at 4 °C. Brain tissues from the peri-lesional cortex were rapidly dissected, weighed, and homogenized in ice-cold assay-specific extraction buffer at a ratio of 1:9 (w/v; 100 mg tissue in 900 μL buffer) to generate 10% tissue homogenates. The homogenates were centrifuged at 12,000 × g for 10 min at 4 °C, and the supernatants were collected for subsequent biochemical assays. Levels of DNA, MPO-DNA complexes, H3cit, H3cit-DNA complexes, MDA, Fe²⁺, and SOD activity were measured in plasma or brain tissue homogenates as indicated. Data obtained from brain tissue homogenates were normalized to total protein concentration determined by a BCA assay. For *in vitro* experiments, HT22 cells were washed with ice-cold PBS, lysed in assay-specific extraction buffer, and centrifuged at 12,000 × g for 10 min at 4 °C. The resulting supernatants were collected for MDA, SOD activity, and Fe²⁺ detection and normalized to total protein content where appropriate. Species-specific ELISA kits were used for human and mouse samples. Detailed information for all ELISA and biochemical assay kits is provided in the **Supplementary Table S2**.

### Western blot analysis

Following euthanasia, mice were transcardially perfused with ice-cold PBS, and brain tissues from the peri-lesional cortex were promptly dissected, with corresponding areas from sham-operated animals serving as controls. Protein lysates of equal amount were denatured in loading buffer at 100 °C for 15 min, separated by 12% SDS-PAGE, and electrophoretically transferred to 0.45 μm polyvinylidene difluoride (PVDF) membranes (Millipore, USA). After blocking, the membranes were incubated overnight at 4 °C with primary antibodies listed in **Supplementary Table S3.** Mouse anti-β-actin (1:5000, Cell Signaling Technology) was used as the loading control for non-histone proteins detected in whole-cell or tissue lysates. For citrullinated histone H3 (H3cit), total histone H3 was used as the normalization control because H3cit represents a post-translationally modified form of histone H3. The same normalization strategy was applied consistently across western blot analyses and figure quantifications. Subsequently, the membranes were washed with TBST and incubated with species-appropriate HRP-conjugated secondary antibodies (1:5000, Cell Signaling Technology) for 1 h at room temperature. Protein bands were visualized using a ChemiDoc Imaging System (Bio-Rad, USA), and band intensities were quantified with ImageJ software (Version 1.46r).

### Histological assessment of neuronal death and damage

To comprehensively assess the extent of neuronal injury following CCI, we employed a multi-faceted histological strategy: Nissl staining for evaluating overall neuronal structural integrity, Fluoro-Jade C (FJC) for labeling degenerating neurons, and terminal deoxynucleotidyl transferase dUTP nick end labeling (TUNEL) staining for detecting apoptotic cell death. For brain tissue preparation, euthanized mice were transcardially perfused with ice-cold PBS followed with 4% paraformaldehyde (PFA). The whole brains were rapidly removed, fixed in 4% PFA at 4°C for 24 h, and coronally sectioned into 10-μm-thick slices using a cryostat (Leica, CM1950, Germany). All subsequent quantitative analyses were performed on these coronal sections encompassing the peri-contusional cortex and hippocampus. Nissl-stained sections were imaged using a bright-field microscope, while FJC- and TUNEL-stained sections were examined under an inverted fluorescence microscope. For all assays, positive cells in both the cortex and hippocampus of each brain were manually counted using ImageJ software (Version 1.46r, Wayne Raband, USA). These analyses were conducted by an investigator blinded to the experimental groups, with data expressed as the number of positive cells per mm². The detailed experimental protocols for each staining are described as follows:

**Nissl staining** was performed to examine the cytoarchitectural integrity and viability of neurons in the contused brain regions after CCI as previously reported^5^. Formaldehyde-fixed specimens were embedded in paraffin and sliced into 8-um thick sections. The sections were deparaffinized with xylene and dehydrated in a graded series of alcohol. The slices were then incubated with Nissl staining solution (Sigma Aldrich) for 30 min at 60 °C. The damaged neurons were characterized by a shrunken cytoplasm and concentrated staining, while the survival neurons were characterized by a large and full soma.

**FJC staining** was conducted to specifically label degenerating neurons during the acute phase post-CCI. Brain sections staining was performed according to the manufacturer's instructions. Briefly, brain sections were rehydrated and incubated with 0.0001% FJC working solution. After rinsing, the slides were dried, cleared in xylene, and coverslipped. FJC-positive neurons—displaying bright fluorescence under green excitation—were visualized and counted in regions of interest using fluorescence microscopy, indicating active neuronal degeneration.

**TUNEL staining** was conducted to detect apoptotic DNA fragmentation in the brain section. To specifically attribute this signal to neurons, the assay was combined with immunofluorescence staining for the neuronal marker NeuN. After overnight incubation with the primary anti-NeuN antibody, the TUNEL reaction procedure was carried out concurrently with the application of fluorescent secondary antibodies the following day, using an *in Situ* Cell Death Detection Kit (Roche, South San Francisco, CA, USA) according to the manufacturer's protocol. Nuclei were then counterstained with 4′,6-diamidino-2-phenylindole (DAPI, Abcam). Apoptotic neurons (NeuN+/TUNEL+) were visualized and counted in regions of interest using fluorescence microscopy.

### Immunofluorescence staining

Following preparation, brain sections were washed with PBS and then permeabilized with 0.1% Triton X-100 (Sigma Aldrich) for 30 minutes, prior to a 1-hour block in 3% BSA at ambient temperature. The samples were then exposed to primary antibodies and maintained at 4 °C overnight. Details regarding the primary antibodies applied in this immunofluorescence assay are provided in **Supplementary Table S4.** Afterward, the sections were treated with species-matched secondary antibodies conjugated to Alexa Fluor (1:500 dilution, Invitrogen, USA) for 1-hour at room temperature. Nuclear staining was performed using DAPI. Imaging was conducted with an inverted fluorescence microscope (Olympus, Japan). For every mouse brain section, three distinct regions along the cortical lesion border within each section were selected and digitized under identical imaging settings. To evaluate neuronal loss, the NeuN immunoreactive area percentage was quantified in three regions—cortex (CTX), hippocampal CA1, and dentate gyrus (DG)—across three coronal sections per animal. Given the known cellular heterogeneity in these regions, area-based quantification was employed instead of direct cell counting^33^, ^41^. Image analysis was performed using ImageJ software (Version 1.46r, Wayne Raband, USA). Briefly, fluorescence images were opened in ImageJ and converted to 8-bit grayscale images. Regions of interest (ROIs) were manually delineated before thresholding. For cortical NeuN analysis, the entire microscopic field along the peri-lesional cortex was defined as the ROI. For hippocampal CA1 and DG analyses, ROIs were selected according to anatomical boundaries identified by NeuN staining and DAPI counterstaining. A fixed positive-staining threshold was established using representative control and injured sections and then applied consistently to all images within the same staining batch. Images were binarized after thresholding, and the NeuN-positive area was automatically measured. The NeuN immunoreactive area percentage was calculated as NeuN-positive area divided by total ROI area × 100%. All image acquisition and quantification were performed by investigators blinded to group allocation.

### Perl's-DAB ferric iron staining

Briefly, the sections were permeabilized with 0.3% Triton X-100 in PBS for 5 min at room temperature. After washing, the slides were incubated in freshly prepared Perl's working solution (5% potassium ferrocyanide mixed 1:1 with 5% hydrochloric acid) at 37 °C for 30 min. The sections were then washed thoroughly and sequentially treated with the incubation solution and the DAB-based enhancement solution provided in the kit, each for 30 min, according to the manufacturer's instructions. Finally, the sections were imaged using a bright-field microscope (Olympus, Japan). Iron accumulation appeared as dark blue to brown granular deposits within the brain tissue.

### HT22 cell culture and treatment

HT22 murine hippocampal neuronal cells, obtained from the Shanghai Institute of Cell Biology (Shanghai, China), were plated in uncoated culture dishes and maintained in high-glucose DMEM (Gibco, USA) supplemented with 10% fetal bovine serum (FBS) and 1% penicillin-streptomycin (Gibco, USA). Cells were cultured at 37 °C in a humidified incubator containing 5% CO₂. The HT22 cell lines used in this study were not passaged more than 15 times.

For NET stimulation, HT22 cells were seeded to reach ~70-80% confluence and then exposed to NET-rich supernatants at the indicated NET-derived DNA concentrations and time points. To digest NET-derived extracellular DNA and disrupt the NET backbone, NET preparations were incubated with deoxyribonuclease I (DNase I, Sigma-Aldrich, USA) at 20 μg/mL for 30 min at 37 °C before being applied to HT22 cells, a concentration and incubation duration commonly used to degrade NET DNA *in vitro* without affecting cell viability in downstream assays^42^. For pharmacological inhibition of ferroptosis, HT22 cells were pre-treated with the ferroptosis inhibitor Liproxstatin-1 (Lip-1; MedChemExpress, USA) at 0.2 μM for 1 h prior to NET exposure, and Lip-1 was maintained in the culture medium throughout the NET stimulation period^43^. To activate AMPK signaling, HT22 cells were pre-treated with the AMPK agonist A-769662 (Selleck, USA) at 50-100 μM for 1-2 h prior to NET stimulation, with A-769662 maintained during subsequent incubations^44^. Vehicle controls (PBS or DMSO at matched final concentrations) were included in all experiments.

### Preparation and quantification of NET-rich supernatants

Neutrophils were isolated from the peripheral blood of euthanized mice using a previously described protocol^44^. Briefly, peripheral blood cells were subjected to hypotonic red blood cell lysis, washed, and incubated sequentially with a Neutrophil Biotin-Antibody Cocktail and MicroBeads (Miltenyi Biotec, Germany). Unlabeled neutrophils were collected as flow-through using a MACS Separator. Purified neutrophils were resuspended in RPMI-1640 medium (Gibco, USA) at 5 × 10⁵ cells/mL and allowed to adhere in 48-well plates at 37 °C for 30 min. Phorbol 12-myristate 13-acetate (PMA) was prepared as a concentrated stock solution in DMSO, aliquoted, protected from light, and freshly diluted in RPMI-1640 medium immediately before each experiment. The final PMA concentration used to induce NET formation was 100 nM, and the working concentration was confirmed by dilution calculation based on the stock concentration and final culture volume. Vehicle control wells received the corresponding final concentration of DMSO. NET formation was induced by stimulation with PMA for 4 h at 37 °C. After stimulation, the culture medium was carefully removed, and adherent NETs were gently washed twice with sterile PBS to remove residual PMA and soluble non-NET components. NETs attached to the culture surface were then released by incubation with the restriction enzyme AluI (4 U/mL; New England Biolabs, USA) for 20 min at 37 °C, following previously published AluI-based NET preparation protocols^45^. The resulting NET-containing supernatants were collected and centrifuged at 500 × g for 5 min at 4 °C to remove residual intact cells and large cellular debris. The clarified NET-rich supernatants were either used immediately or stored at -80 °C. NET preparations were quantified based on extracellular DNA content using a PicoGreen dsDNA assay kit according to the manufacturer's instructions (Invitrogen, USA), following previously published and validated NET quantification protocols^45^, ^46^. NET doses were expressed as NET-derived DNA concentrations (1-4 μg/mL) and applied to HT22 cells for downstream assays.

### STING knockdown and pharmacological STING inhibition in HT22 cells

For STING silencing, small interfering RNAs specifically targeting mouse STING (si-STING), together with a nonspecific scrambled siRNA as a negative control (si-NC), were obtained from Hanbio (Shanghai, China). HT22 cells were transiently transfected with siRNAs using Lipofectamine™ RNAiMAX (Invitrogen, USA) following the manufacturer's recommended protocol. The efficiency of STING knockdown was confirmed by western blot analysis before subsequent experimental procedures. The si-STING sequences (sense: 5′-GGAGCCGAAGACUGUGUACAUTT-3′; antisense: 5′-AUGUACAGUCUUCGGCUCCTT-3′) were designed by Hanbio to target the mouse Tmem173 (STING) coding region and validated for specificity. The scrambled negative control siRNA (si-NC) was provided by the same vendor. To pharmacologically inhibit endogenous STING signaling *in vitro*, HT22 cells were pre-treated with the STING inhibitor C-176 (Selleck, USA) at the indicated working concentration for 1 h before NET stimulation, and C-176 was maintained during subsequent incubations. Matched vehicle controls were included.

### Cell viability, LDH release, and SYTOX Green staining

Cell viability was assessed using a Cell Counting Kit-8 (CCK-8, Beyotime, China) to evaluate time-dependent changes in metabolic activity following NET exposure. Briefly, HT22 cells were seeded into 96-well plates and subjected to the indicated treatments. At the specified time points (e.g., 12, 24, and 36 h), CCK-8 reagent was added to each well and incubated at 37 °C for 2 h. The optical density (OD) at 450 nm was measured using a microplate reader. Cell viability was expressed as absolute OD values as indicated.

Cell injury-associated cytotoxicity was quantified by measuring lactate dehydrogenase (LDH) released into the culture supernatant using an LDH Cytotoxicity Assay Kit (Beyotime, China), according to the manufacturer's instructions. LDH release was expressed as fold change relative to control conditions.

To visualize membrane-compromised and dead cells, HT22 cells subjected to NET stimulation for 24 h were gently washed with phosphate-buffered saline (PBS). Subsequently, SYTOX™ Green nucleic acid stain (Invitrogen, USA) diluted 1:2000 in serum-free medium was added to each well according to the manufacturer's instructions. Cells were incubated at 37 °C for 15 min in the dark, after which the staining solution was removed and cells were gently rinsed with PBS. The cells were then immediately examined using a fluorescence microscope. SYTOX-positive dead cells were quantified and expressed as the percentage of total cells.

### Statistical analysis

Normality of the data was assessed using the Shapiro-Wilk test. Data are presented as mean ± SD with individual data points. All analyses were performed by an experimenter blinded to the group assignments using GraphPad Prism 8.1.2 (GraphPad Software, San Diego, CA, USA). For comparisons among multiple groups at a single time point, one-way ANOVA followed by Tukey's post hoc test was applied. Repeated measurements, such as neurological deficit scores over time, were analyzed using two-way repeated-measures ANOVA to assess the effects of group, time, and their interaction, followed by Tukey's post hoc test. Comparisons between two independent groups were performed using the Mann-Whitney U test. All statistical tests were two-tailed, and P < 0.05 was considered statistically significant.

## Results

### Increased peripheral NET formation correlates with peripheral neuronal injury biomarkers and neurological severity in patients with TBI

**Figure 1:**
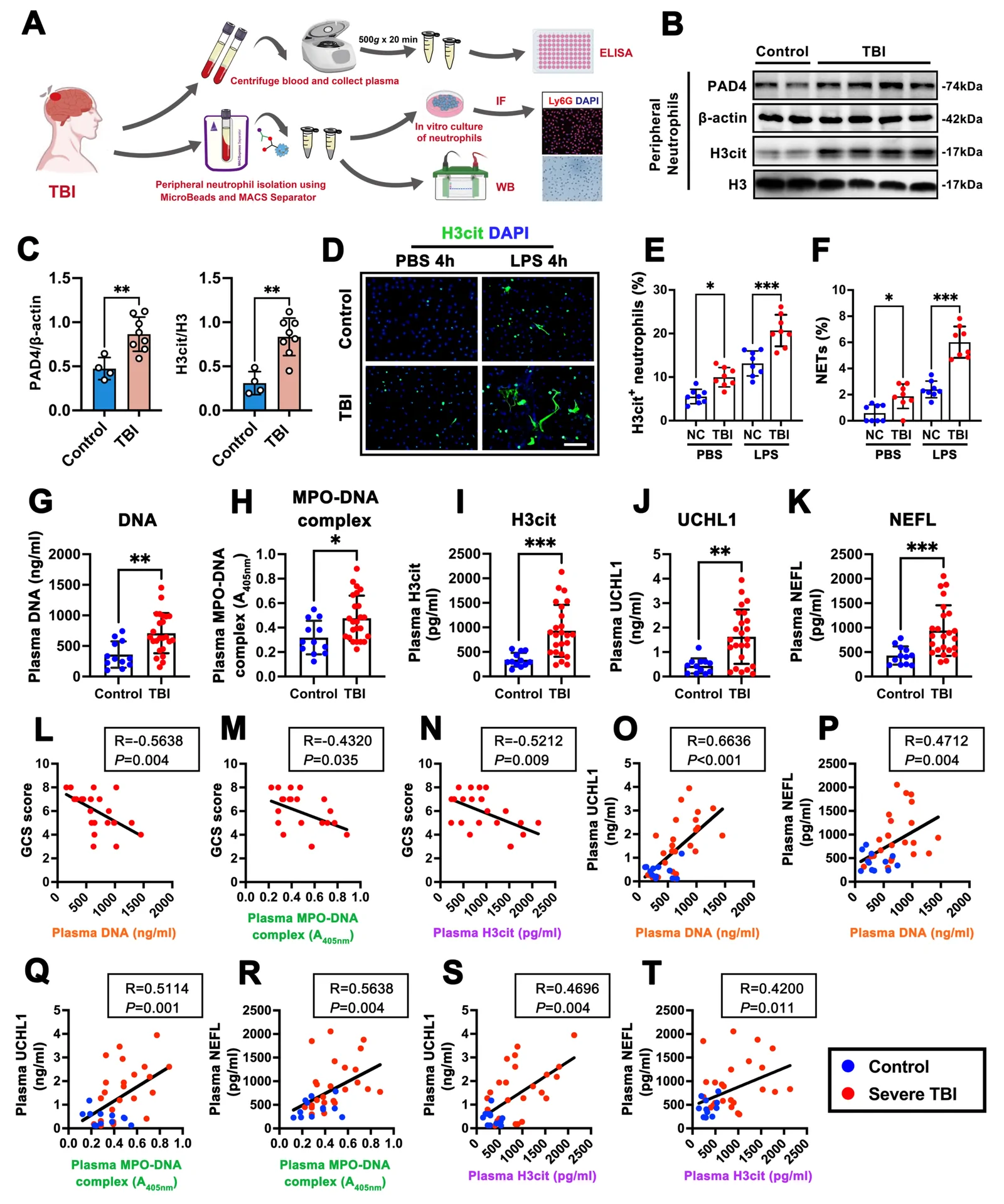
** Enhanced NET formation in peripheral neutrophils is associated with increased neurological severity after traumatic brain injury. A** Schematic illustration of peripheral blood collection, plasma isolation, neutrophil purification using MicroBeads and MACS, followed by *in vitro* neutrophil culture and downstream analyses. **B, C** Representative western blot bands (B) and densitometric quantification (C) of peptidylarginine deiminase 4 (PAD4; normalized to β-actin) and citrullinated histone H3 (H3cit; normalized to histone H3) in peripheral neutrophils from control subjects (n = 4) and patients with severe TBI (n = 8). **P < 0.01. **D-F** Representative immunofluorescence images (D) and quantitative analyses of H3cit-positive neutrophils (E) and NET formation (F) following PBS or LPS stimulation for 4 h. Nuclei were stained with DAPI. Scale bar = 50 μm. *P < 0.05, ***P < 0.001, n = 8 per group. **G-I** Plasma levels of DNA (G), MPO-DNA complexes (H), and H3cit (I) in control subjects (n = 12) and patients with severe TBI (n = 24). **J, K** Plasma neuronal injury biomarkers UCHL1 (J) and NEFL (K) in control subjects (n = 12) and patients with severe TBI (n = 24). **L-N** Correlation analyses between Glasgow Coma Scale (GCS) scores and plasma DNA (L), MPO-DNA complexes (M), or H3cit levels (N) in patients with severe TBI (n = 24). **O-T** Correlation analyses between plasma NET-associated markers (DNA, MPO-DNA complexes, and H3cit) and neuronal injury biomarkers (UCHL1 or NEFL) across all subjects (control subjects, n = 12; patients with severe TBI, n = 24). Pearson's correlation coefficients (R) and P values are shown as insets.

To determine whether TBI induces systemic NET formation, we first examined peripheral neutrophils and plasma collected from TBI patients and healthy controls. Western blot analysis revealed markedly increased PAD4 expression and elevated levels of citrullinated histone H3 (H3cit), a PAD4-mediated histone modification that promotes chromatin decondensation and serves as a marker of NET formation, in neutrophils isolated from TBI patients compared with controls (Fig. 1B, C). Immunofluorescence staining further confirmed that the proportion of H3cit⁺ neutrophils was significantly higher in peripheral blood smears from TBI patients under both basal and LPS-stimulated conditions (Fig. 1D, E). Consistently, NET-positive neutrophils showed a prominent increase in the TBI group (Fig. 1F).

To further verify the association between NET formation and secondary neuronal injury after TBI, we quantified circulating NET-related components and neuronal injury biomarkers using ELISA and collected the corresponding GCS scores from the same cohort of patients. Plasma DNA, MPO-DNA complexes, and H3cit levels were all significantly elevated in TBI patients compared with healthy controls (Fig. 1G-I). In parallel, plasma level of UCHL1 and NEFL, two established indicators of neuronal injury, were markedly increased after TBI (Fig. 1J, K). Correlation analyses revealed significant negative associations between GCS scores and plasma DNA, MPO-DNA complexes, and H3cit levels (Fig. 1L-N), indicating that higher NET burden was linked to worse neurological status. Moreover, plasma DNA levels showed strong positive correlations with UCHL1 and NEFL (Fig. 1O, P). MPO-DNA complexes also positively correlated with UCHL1 and NEFL (Fig. 1Q, R), and consistent results were observed for H3cit, which positively correlated with both neuronal injury markers (Fig. 1S, T). These findings indicate that elevations in circulating NET markers closely reflect neurological severity and neuronal injury following TBI.

### Intracerebral NET accumulation is accompanied by neuronal ferroptosis in human TBI brain tissue

**Figure 2:**
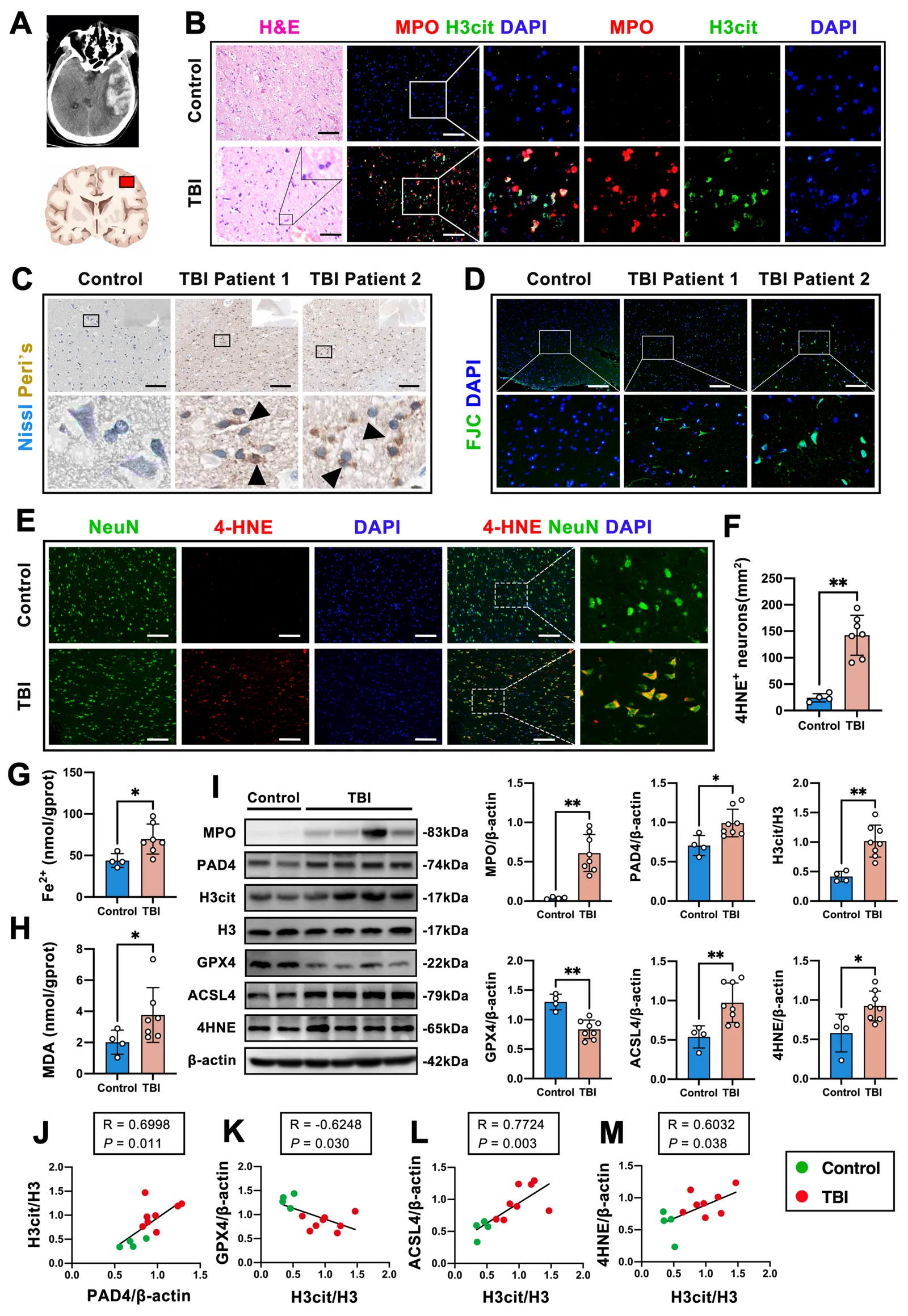
** NET deposition is associated with ferroptosis-associated neuronal injury in the injured human cortex after traumatic brain injury. A** Schematic illustration and computed tomography (CT) image indicating the contusional cortical region used for histological analyses (red box). **B** Representative H&E staining and immunofluorescence staining for neutrophils (MPO, red), NET marker H3cit (green), and nuclei (DAPI, blue) in cortical tissues from control subjects (n = 4) and patients with TBI (n = 8). Scale bar = 100 μm. **C** Representative Nissl and Perl's staining of cortical sections from control subjects (n = 4) and patients with TBI (n = 8), showing neuronal morphology (Nissl, blue) and iron deposition visualized as yellow to brown granular deposits in peri-contusional cortex. Scale bar = 100 μm. **D** Representative Fluoro-Jade C (FJC) staining of degenerating neurons (green) and nuclei (DAPI, blue) in cortical tissues from control subjects (n = 4) and patients with TBI (n = 8). Scale bar = 200 μm. **E, F** Representative immunofluorescence staining and quantitative analyses of 4-HNE-positive neurons in cortical tissues from control subjects (n = 4) and patients with TBI (n = 8). Scale bar = 100 μm. **P < 0.01. **G, H** Quantitative analyses of Fe²⁺ (G) and malondialdehyde (MDA) (H) levels in cortical tissues from control subjects (n = 4) and patients with TBI (n = 8). *P < 0.05. **I** Representative western blot bands and densitometric quantification of MPO, PAD4, H3cit, GPX4, ACSL4, and 4-HNE in cortical tissues from control subjects (n = 4) and patients with TBI (n = 8). Histone H3 and β-actin were used as loading controls. *P < 0.05, **P < 0.01. **J-M** Correlation analyses between PAD4/β-actin and H3cit/H3 (J), H3cit/H3 and GPX4/β-actin (K), H3cit/H3 and ACSL4/β-actin (L), or H3cit/H3 and 4-HNE/β-actin (M). Pearson's correlation coefficients (R) and P values are shown as insets. Green dots indicate control subjects, and red dots indicate patients with TBI.

To investigate whether NET formation also occurs within the injured brain, we examined contusional cortical tissue surgically resected from patients with TBI (Fig. 2A). Immunofluorescence staining further demonstrated abundant MPO⁺/H3cit⁺ neutrophils forming NET-like structures throughout the injured parenchyma, whereas these signals were nearly absent in control samples (Fig. 2B), confirming pronounced intracerebral NET accumulation after TBI. To determine whether intracerebral NET deposition is associated with neuronal ferroptosis, we next performed Nissl and Perl's Prussian blue staining. TBI cortical tissue showed a marked increase in Prussian blue-positive neurons compared with control brains, indicating pronounced iron overload in neurons after injury (Fig. 2C). Consistently, FJC staining revealed a substantial increase in FJC-positive degenerating neurons in the contusional cortex of TBI patients (Fig. 2D). Further double immunofluorescence staining showed that TBI cortical tissue exhibited robust 4-HNE accumulation in neurons, which was rarely observed in control tissue (Fig. 2E), and quantification confirmed a significant increase in 4-HNE-positive neurons in TBI samples (Fig. 2F). Biochemical measurements further supported ferroptotic lipid peroxidation, as Fe²⁺ and MDA levels were significantly elevated in TBI cortical lysates (Fig. 2G, H).

To further characterize molecular features of NET formation and ferroptosis after TBI, we performed western blot analysis of brain tissue. TBI samples exhibited increased levels of myeloperoxidase (MPO), PAD4, H3cit, ACSL4, and 4-HNE, alongside a marked reduction in GPX4 expression, compared with control cortex (Fig. 2I). Finally, correlation analyses revealed that H3cit expression was positively correlated with PAD4 levels (Fig. 2J), negatively correlated with GPX4 (Fig. 2K), and positively correlated with ACSL4 and 4-HNE expression (Fig. 2L, M), indicating that intracerebral NET formation is closely linked to ferroptosis-associated neuronal injury. Together, these findings demonstrate that NET accumulation occurs within the injured human brain following TBI and is accompanied by pronounced neuronal ferroptosis-related pathological changes.

### PAD4-dependent NET formation is induced after TBI and contributes to neurological dysfunction

To determine whether NET formation is dynamically induced after traumatic brain injury, we first examined the temporal kinetics of neutrophil recruitment and NET-related markers in a mouse TBI model. Immunofluorescence staining showed a marked increase in MPO-positive neutrophils in the peri-lesional cortex after TBI, with the most prominent accumulation observed during the acute phase (Supplementary Fig. S1A). Western blot analysis further revealed increased MPO and H3cit expression in injured brain tissue over the early post-injury period (Supplementary Fig. S1B-D). Consistently, double immunofluorescence staining demonstrated accumulation of MPO⁺H3cit⁺ cells in the peri-lesional cortex at 1 and 3 days post-injury, indicating enhanced intracerebral NET formation (Supplementary Fig. S1E). In parallel, enhanced NET formation was also observed in the peripheral compartment, as evidenced by increased PAD4 and H3cit expression in circulating neutrophils and elevated plasma levels of cell-free DNA, H3cit, MPO-DNA complexes, and H3cit-DNA complexes after TBI (Supplementary Fig. S1F-M).

**Figure 3:**
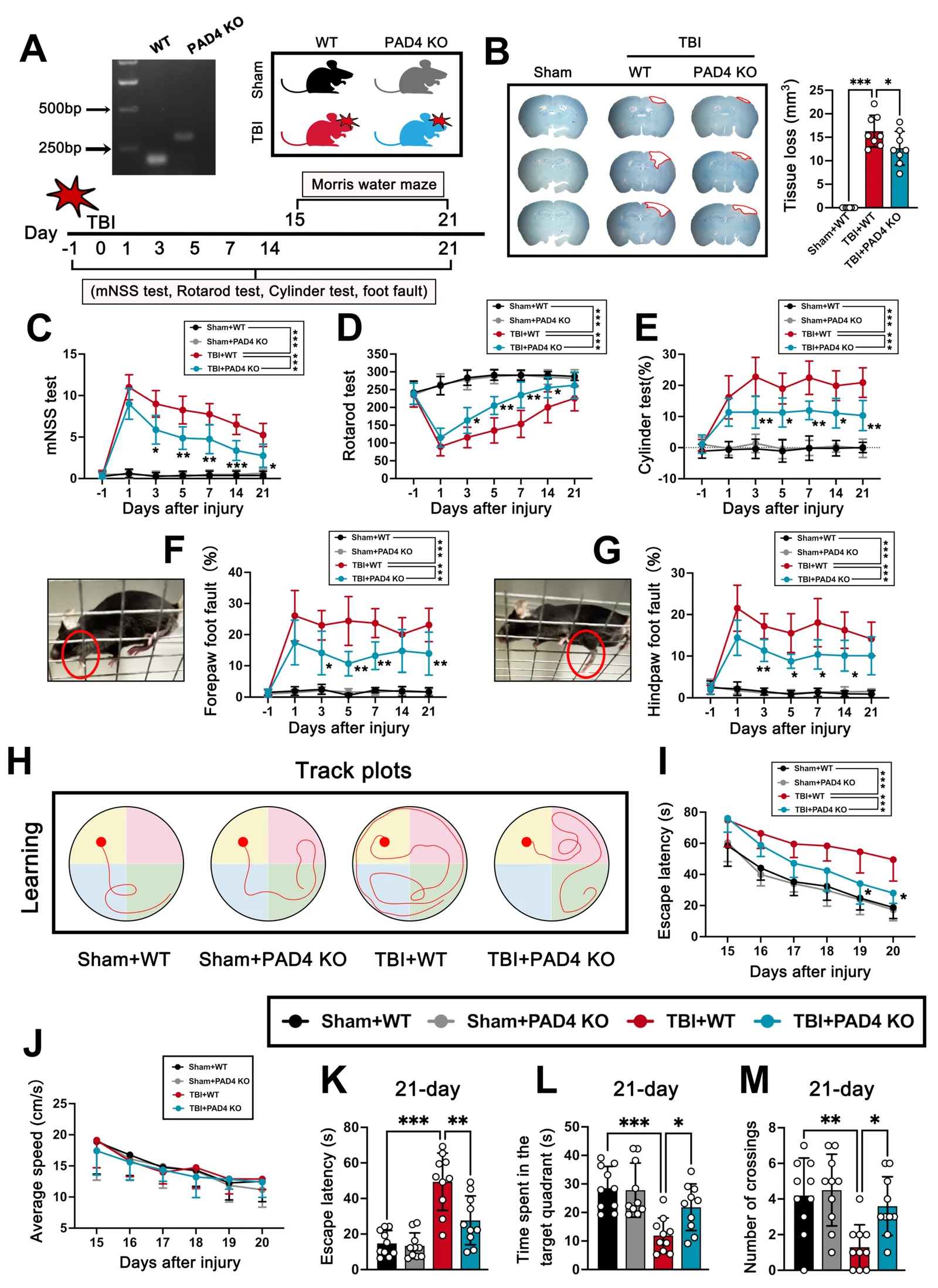
** PAD4 deficiency attenuates tissue loss and improves neurological outcomes after traumatic brain injury. A** Representative genotyping of wild-type (WT) and PAD4 knockout (PAD4 KO) mice and schematic illustration of the experimental design, including behavioral assessments and Morris water maze testing following TBI. **B** Representative images of coronal brain sections and quantitative analyses of tissue loss volume in Sham + WT, TBI + WT, and TBI + PAD4 KO mice. ***P < 0.001, *P < 0.05, n = 8 per group. **C-G** Neurological and motor function assessments, including modified neurological severity score (mNSS) (C), rotarod test (D), cylinder test (E), forepaw foot fault test (F), and hindpaw foot fault test (G), performed at the indicated time points after injury in Sham + WT, Sham + PAD4 KO, TBI + WT, and TBI + PAD4 KO mice. *P < 0.05, **P < 0.01, ***P < 0.001, n = 10 per group. **H** Representative swimming track plots during the learning phase of the Morris water maze in the indicated groups. **I** Escape latency during the learning phase of the Morris water maze from days 15 to 20 after injury. *P < 0.05, **P < 0.01, ***P < 0.001, n = 10 per group. **J** Average swimming speed during the Morris water maze learning phase. No significant differences were detected among groups by two-way repeated-measures ANOVA followed by Tukey's post hoc test; n = 10 per group. **K-M** Probe trial analyses at day 21 after injury, including escape latency (K), time spent in the target quadrant (L), and number of platform crossings (M). *P < 0.05, **P < 0.01, ***P < 0.001, n = 10 per group.

Given the essential role of PAD4 in histone citrullination and NET formation, we next used PAD4 knockout (PAD4 KO) mice to assess the functional contribution of NETs to TBI pathology (Fig. 3A). Compared with wild-type (WT) mice, PAD4 KO mice exhibited significantly reduced tissue loss at 3 days post-injury (Fig. 3B). Consistent with attenuated tissue damage, PAD4 deficiency markedly improved neurological recovery, as indicated by lower mNSS scores, enhanced motor coordination in the rotarod test, reduced forelimb asymmetry in the cylinder test, and fewer forepaw and hindpaw foot-fault errors (Fig. 3C-G). Long-term cognitive function was further evaluated using the Morris water maze. PAD4 KO mice displayed improved spatial learning, with shorter escape latencies during training (Fig. 3H, I). Importantly, average swimming speed during the acquisition phase did not differ significantly among groups (Fig. 3J), indicating that differences in Morris water maze performance were unlikely to be attributable to altered swimming ability or motor impairment. In the probe trial, PAD4 KO mice showed superior memory performance, as reflected by shorter escape latency, increased time spent in the target quadrant, and a greater number of platform crossings (Fig. 3K-M). Together, these results demonstrate that PAD4-dependent NET formation is robustly induced after TBI and contributes to tissue damage and both short- and long-term neurological dysfunction.

### PAD4 deficiency protects against neuronal loss after TBI

**Figure 4:**
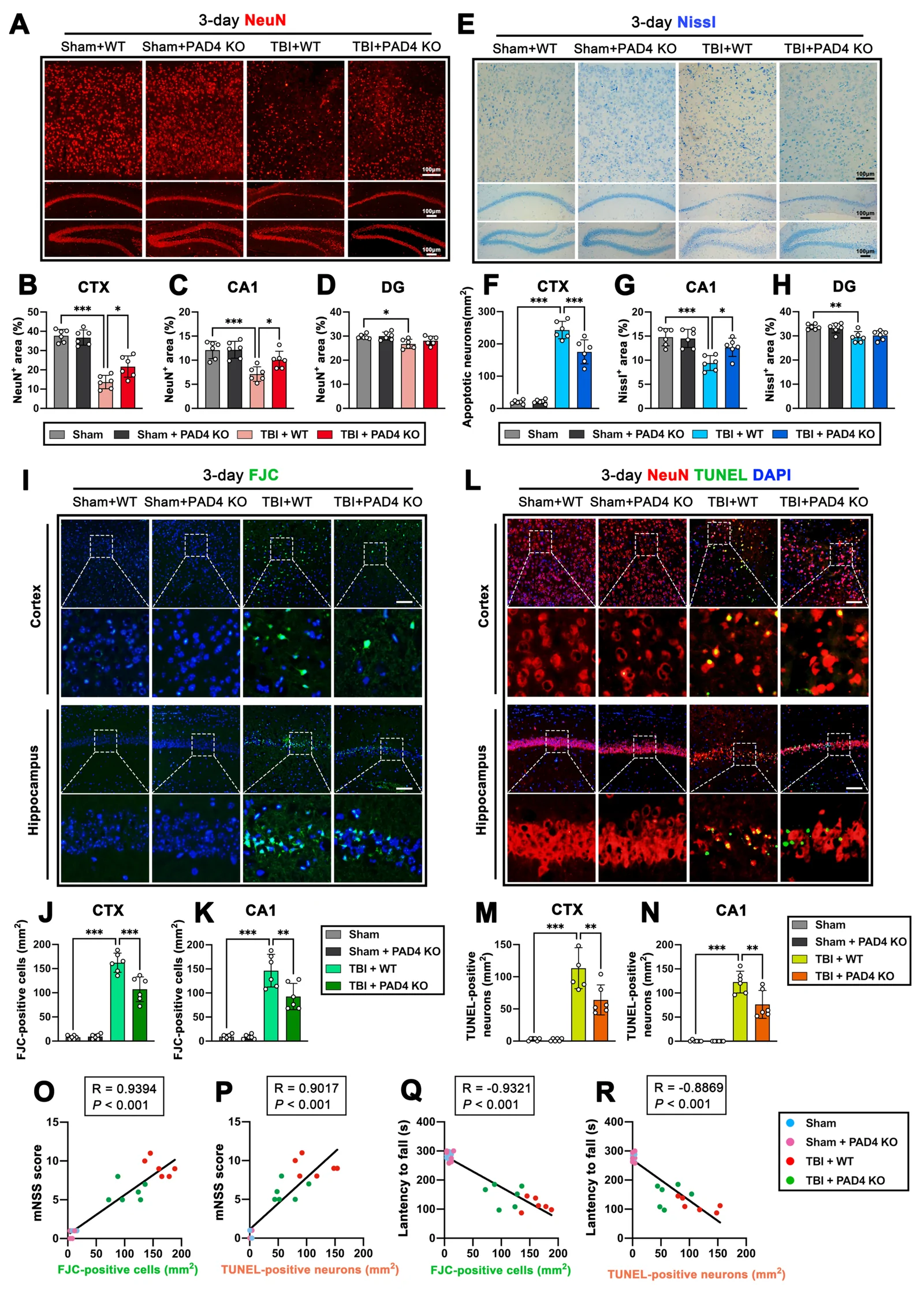
** PAD4 deficiency attenuates neuronal death at 3 days after traumatic brain injury. A** Representative images of NeuN immunostaining in the ipsilateral cortex (CTX), CA1, and dentate gyrus (DG) regions at 3 days after TBI in Sham + WT, Sham + PAD4 KO, TBI + WT, and TBI + PAD4 KO mice. **B-D** Quantitative analyses of NeuN-immunopositive area in the ipsilateral CTX (B), CA1 (C), and DG (D) regions at 3 days after TBI. *P < 0.05, ***P < 0.001, n = 6 per group. **E** Representative images of Nissl staining in the ipsilateral CTX, CA1, and DG regions at 3 days after TBI. **F-H** Quantitative analyses of Nissl-positive area in the ipsilateral CTX (F), CA1 (G), and DG (H) regions at 3 days after TBI. *P < 0.05, **P < 0.01, ***P < 0.001, n = 6 per group. **I** Representative images of Fluoro-Jade C (FJC, green) staining in the ipsilateral cortex and hippocampus at 3 days after TBI. Nuclei were stained with DAPI (blue). Scale bar = 100 μm. **J, K** Quantitative analyses of FJC-positive cells in the ipsilateral CTX (J) and CA1 (K) regions at 3 days after TBI. **P < 0.01, ***P < 0.001, n = 6 per group. **L** Representative images of TUNEL (green) co-localization with neurons (NeuN, red) in the ipsilateral cortex and hippocampus at 3 days after TBI. Nuclei were stained with DAPI (blue). Scale bar = 100 μm. **M, N** Quantitative analyses of TUNEL-positive neurons in the ipsilateral CTX (M) and CA1 (N) regions at 3 days after TBI. **P < 0.01, ***P < 0.001, n = 6 per group. **O-R** Correlation analyses between mNSS score and FJC-positive cells (O) or TUNEL-positive neurons (P), and between latency to fall and FJC-positive cells (Q) or TUNEL-positive neurons (R). Pearson's correlation coefficients (R) and P values are shown as insets.

To assess whether PAD4 deficiency alleviates neuronal damage following TBI, we first evaluated neuronal survival in the ipsilateral cortex (CTX) and hippocampal subregions at 3 days post-injury. NeuN immunofluorescence revealed substantial neuronal loss in CTX, CA1, and DG after TBI, whereas PAD4 KO mice exhibited significantly preserved NeuN⁺ neuronal area in both CTX and CA1), with only minor changes observed in the DG (Fig. 4A-D). These findings indicate that PAD4 deficiency confers robust neuroprotection in the brain regions most vulnerable to acute TBI-induced damage. To further verify structural neuronal integrity, Nissl staining was performed in corresponding regions (Fig. 4E). Consistent with the NeuN results, TBI caused marked neuronal loss in CTX and CA1, while PAD4 KO significantly reduced Nissl-positive neuronal damage in these areas (Fig. 4F-H). The DG displayed comparatively mild injury and no significant rescue in PAD4 KO mice. Given that the strongest protective effects were observed in CTX and CA1, subsequent analyses focused on these regions. FJC staining demonstrated a pronounced increase in degenerating neurons in TBI WT mice, whereas PAD4 KO markedly diminished FJC-positive cells in both CTX and CA1 (Fig. 4I-K). Similarly, TUNEL staining revealed abundant apoptotic neurons after TBI, and PAD4 deficiency significantly reduced TUNEL-positive neuronal death in the same regions (Fig. 4L-N). These data suggest that PAD4-dependent NET formation promotes both degenerative and apoptotic neuronal injury following TBI. Finally, correlation analyses demonstrated that neuronal cell death strongly predicted neurological impairment. Both FJC-positive and TUNEL-positive neurons showed strong positive correlations with mNSS scores (Fig. 4O-P) and strong negative correlations with rotarod latency (Fig. 4Q-R). These associations highlight the functional relevance of PAD4-mediated neuronal injury and further support the neuroprotective role of PAD4 deficiency after TBI.

### NET-derived DNA directly induces ferroptosis-associated neuronal injury *in vitro*

**Figure 5:**
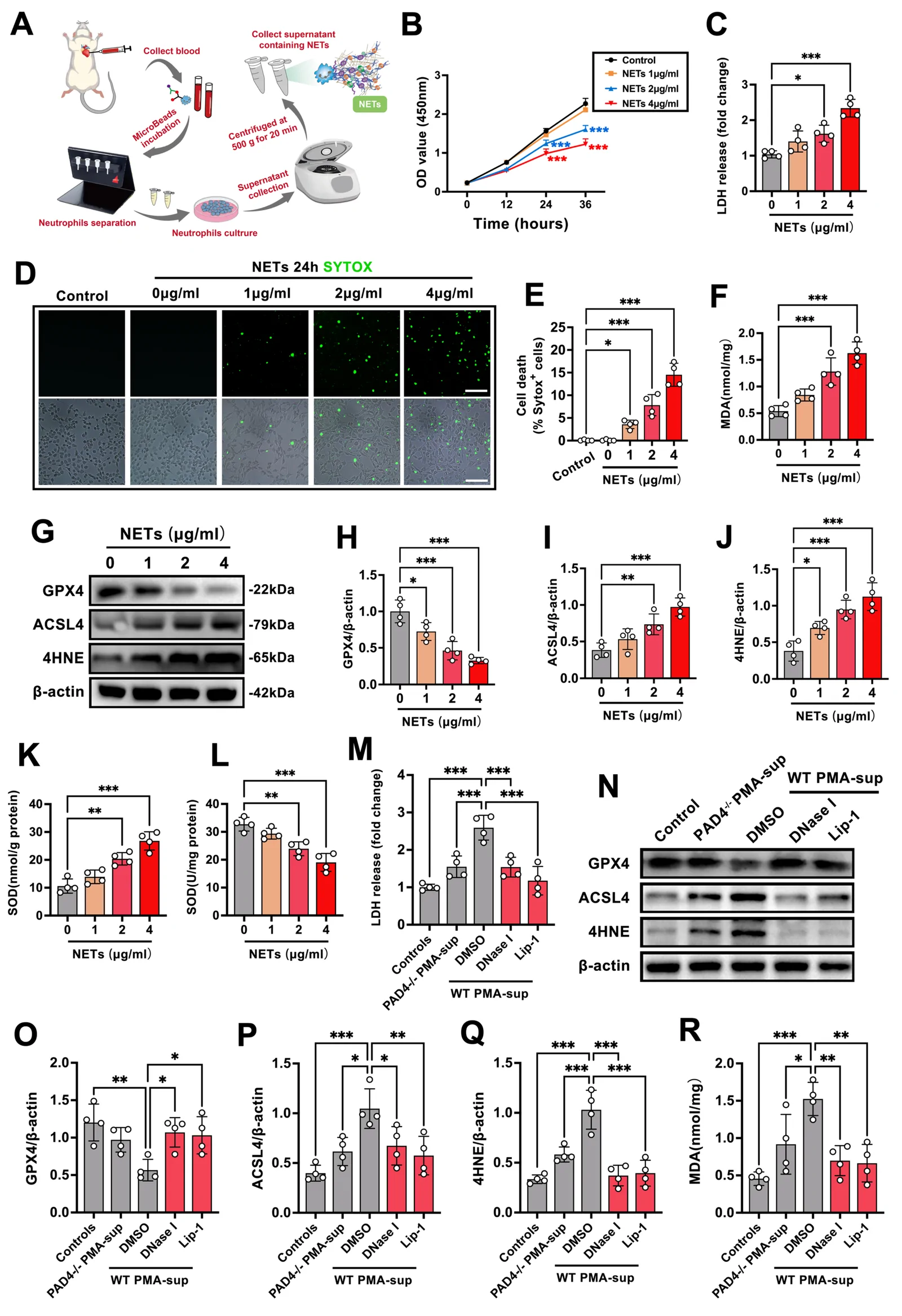
** NETs directly induce ferroptosis-associated neuronal injury *in vitro* in a DNA-dependent manner.** A Schematic illustration of mouse neutrophil isolation, PMA-induced NET generation, NET-rich supernatant collection, and subsequent stimulation of HT22 cells. **B** CCK-8 assays showing time-dependent changes in HT22 cell viability after treatment with increasing concentrations of NET-derived DNA. ***P < 0.001, n = 4 per group. **C** Quantification of lactate dehydrogenase (LDH) release in HT22 cells treated with different concentrations of NETs for 24 h. *P < 0.05, ***P < 0.001, n = 4 per group. **D** Representative SYTOX Green staining images of HT22 cells after 24 h exposure to NETs. Scale bar = 100 μm. **E** Quantitative analysis of SYTOX Green-positive cell death. *P < 0.05, ***P < 0.001, n = 4 per group. **F** Quantification of malondialdehyde (MDA) levels in NET-treated HT22 cells. ***P < 0.001, n = 4 per group. **G** Representative western blot bands showing GPX4, ACSL4, and 4-HNE expression in HT22 cells treated with increasing concentrations of NETs. **H-J** Densitometric analyses of GPX4 (H), ACSL4 (I), and 4-HNE (J) expression. β-actin was used as a loading control. *P < 0.05, **P < 0.01, ***P < 0.001, n = 4 per group. **K, L** Quantification of intracellular Fe²⁺ levels (K) and superoxide dismutase (SOD) activity (L) in NET-treated HT22 cells. **P < 0.01, ***P < 0.001, n = 4 per group. **M** Quantification of LDH release in HT22 cells treated with supernatants from PMA-stimulated PAD4-/- neutrophils or WT neutrophils, with or without DNase I or Liproxstatin-1 (Lip-1). ***P < 0.001, n = 4 per group. **N** Representative western blot bands showing GPX4, ACSL4, and 4-HNE expression under the indicated conditions. **O-Q** Densitometric analyses of GPX4 (O), ACSL4 (P), and 4-HNE (Q) expression. β-actin was used as a loading control. *P < 0.05, **P < 0.01, ***P < 0.001. **R** Quantification of MDA levels under the indicated conditions. *P < 0.05, **P < 0.01, ***P < 0.001, n = 4 per group.

To determine whether NETs directly induce ferroptosis-associated neuronal injury, mouse neutrophils were stimulated with PMA to generate NETs, and NET-rich supernatants were collected after removal of intact cells and debris (Fig. 5A). NET preparations were quantified based on extracellular DNA content and applied to HT22 neurons at increasing concentrations. NET exposure reduced neuronal viability in a concentration-dependent manner, with significant decreases observed at higher NET-derived DNA levels (2-4 μg/mL) over 24-36 h, as assessed by CCK-8 assays (Fig. 5B). Consistently, LDH release assays revealed a corresponding increase in neuronal membrane damage following NET treatment (Fig. 5C). SYTOX Green staining further demonstrated a progressive increase in neuronal death with increasing NET concentrations (Fig. 5D, E). In parallel, NET exposure significantly elevated MDA levels, indicating enhanced lipid peroxidation (Fig. 5F). Western blot analysis revealed a ferroptosis-associated protein signature, characterized by reduced GPX4 expression and increased ACSL4 and 4-HNE levels in NET-treated neurons (Fig. 5G-J). Given that iron accumulation and impaired antioxidant capacity are hallmarks of ferroptosis, we next examined intracellular iron and antioxidant activity. NET treatment induced a dose-dependent increase in intracellular Fe²⁺ levels and a concomitant reduction in SOD activity (Fig. 5K, L), further supporting the induction of ferroptotic stress. To distinguish NET-dependent neurotoxicity from nonspecific soluble factors released by PMA-activated neutrophils, we further compared supernatants collected from PMA-stimulated WT and PAD4-/- neutrophils. Although both preparations were derived from PMA-activated neutrophils, PAD4-/- PMA-supernatants induced markedly lower LDH release than WT PMA-supernatants in HT22 cells (Fig. 5M). Consistently, PAD4-/- PMA-supernatants caused weaker ferroptosis-associated molecular changes, as evidenced by preserved GPX4 expression and reduced ACSL4 and 4-HNE accumulation compared with WT PMA-supernatants (Fig. 5N-Q). These findings indicate that PAD4-dependent NET formation is a major contributor to PMA-neutrophil supernatant-induced neuronal ferroptotic injury. Moreover, DNase I digestion of WT PMA-supernatants significantly attenuated LDH release, restored GPX4 expression, reduced ACSL4 and 4-HNE accumulation, and lowered MDA levels (Fig. 5M-R). The ferroptosis inhibitor Liproxstatin-1 (Lip-1) produced comparable protective effects, supporting the involvement of ferroptotic lipid peroxidation in NET-induced neuronal injury. Collectively, these results demonstrate that PMA-activated neutrophil supernatants induce ferroptosis-associated lipid peroxidation and neuronal injury primarily through a PAD4/NET-dependent and extracellular DNA-dependent mechanism, and that enzymatic degradation of NET-derived DNA or pharmacological inhibition of ferroptosis effectively mitigates NET-mediated neurotoxicity.

### NET-derived DNA activates neuronal STING signaling and promotes ferroptosis after TBI

**Figure 6:**
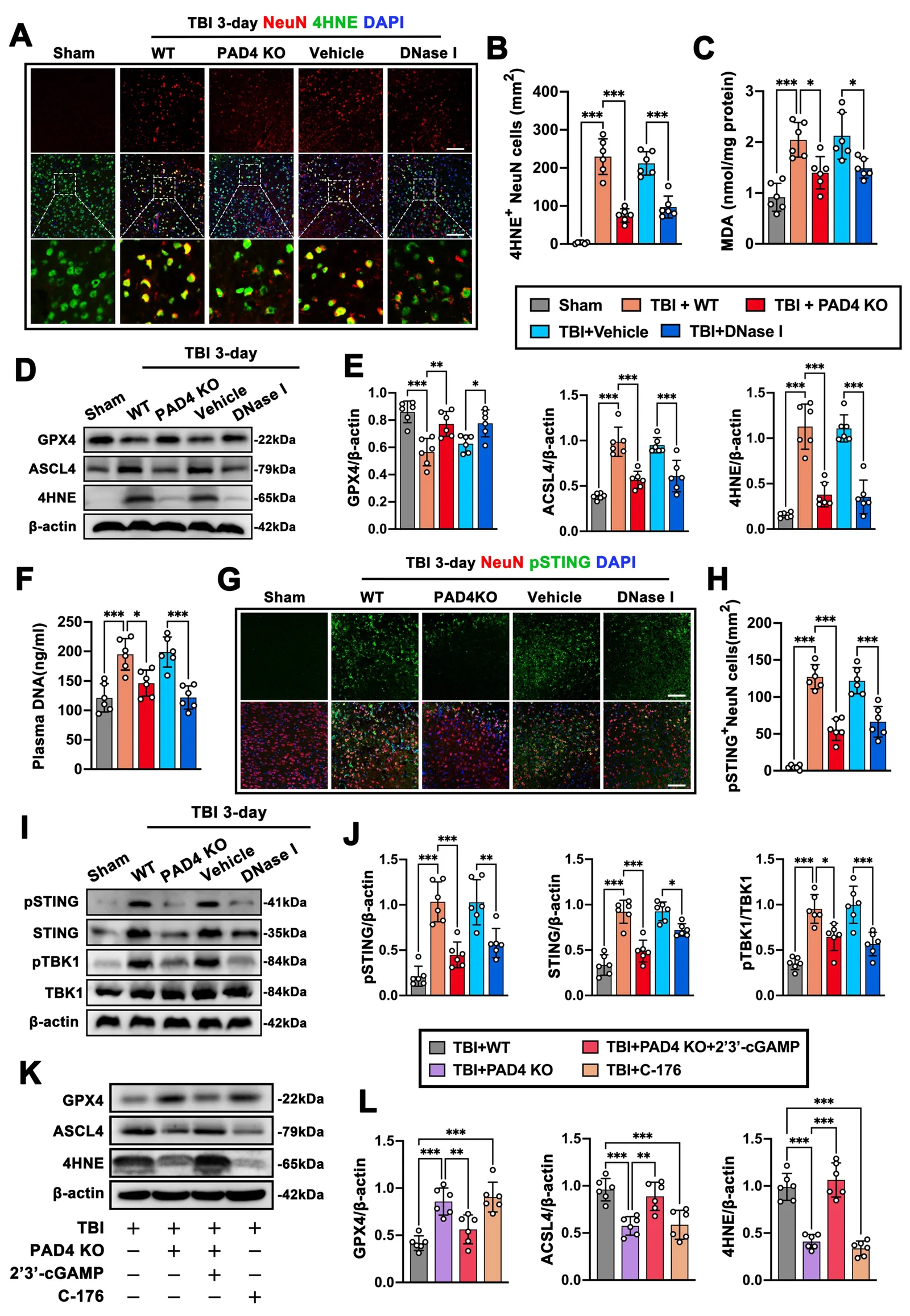
** NET-derived DNA activates neuronal STING signaling and promotes ferroptosis-associated neuronal injury after TBI. A, B** Representative immunofluorescence images (A) and quantitative analyses (B) of NeuN (red) and 4-HNE (green) co-staining in the ipsilateral cortex at 3 days after TBI in Sham, TBI + WT, TBI + PAD4 KO, TBI + Vehicle, and TBI + DNase I-treated mice. Nuclei were stained with DAPI (blue). Insets show higher magnification views. Scale bar = 100 μm. ***P < 0.001, n = 6 per group. **C** Quantitative analyses of cortical MDA levels at 3 days after TBI. *P < 0.05, ***P < 0.001, n = 6 per group. **D, E** Representative western blot bands (D) and densitometric quantification (E) of GPX4, ACSL4, and 4-HNE expression in cortical tissues at 3 days after TBI. β-actin was used as a loading control. *P < 0.05, **P < 0.01, ***P < 0.001, n = 6 per group. **F** Plasma DNA levels at 3 days after TBI. *P < 0.05, ***P < 0.001, n = 6 per group. **G, H** Representative immunofluorescence staining (G) and quantitative analyses (H) of phosphorylated STING (pSTING)-positive neurons in the ipsilateral cortex at 3 days after TBI. Scale bar = 100 μm. ***P < 0.001, n = 6 per group. **I, J** Representative western blot bands (I) and densitometric quantification (J) of pSTING, STING, pTBK1, and TBK1 expression in cortical tissues at 3 days after TBI. β-actin was used as a loading control. *P < 0.05, **P < 0.01, ***P < 0.001, n = 6 per group. **K, L** Representative western blot bands (K) and densitometric quantification (L) of GPX4, ACSL4, and 4-HNE expression in PAD4 KO mice treated with 2′3′-cGAMP or the STING inhibitor C-176 at 3 days after TBI. β-actin was used as a loading control. **P < 0.01, ***P < 0.001, n = 6 per group.

To determine whether NET-derived DNA contributes to neuronal ferroptosis after TBI, we first examined neuronal lipid peroxidation in the peri-contusional cortex. Immunofluorescence staining revealed a marked increase in 4-HNE⁺ NeuN⁺ neurons in TBI WT mice, whereas both PAD4 KO and DNase I treatment significantly reduced neuronal 4-HNE accumulation (Fig. 6A-B). Consistently, MDA levels were markedly elevated after TBI but were significantly attenuated by PAD4 deletion or NET degradation (Fig. 6C). Western blot analysis further confirmed the ferroptotic signature: TBI WT mice displayed decreased GPX4 expression and increased ACSL4 and 4-HNE levels, while PAD4 KO and DNase I restored GPX4 and reduced ACSL4/4-HNE expression (Fig. 6D, E). These results indicate that NET formation may contribute to neuronal ferroptosis during acute phase after TBI.

Because extracellular DNA is the major structural component of NETs, we next quantified circulating NET-derived DNA. Plasma DNA levels were markedly elevated following TBI, and both PAD4 KO and DNase I significantly reduced this increase (Fig. 6F), suggesting that NET-derived DNA may function as an upstream danger signal after TBI. Given that cytosolic DNA activates the cGAS-STING pathway, we assessed neuronal STING activation. Immunofluorescence showed strong phosphorylated STING (pSTING) accumulation in NeuN⁺ neurons of TBI WT mice, whereas PAD4 KO and DNase I markedly reduced neuronal pSTING levels (Fig. 6G-H). Western blot analysis further confirmed that TBI induced robust increases in pSTING, total STING, and phosphorylated TANK-binding kinase 1 (pTBK1), all of which were significantly suppressed by PAD4 deletion or DNase I treatment (Fig. 6I-J). These findings demonstrate that NET-derived DNA serves as a principal trigger of neuronal STING activation after TBI. To further confirm the role of STING signaling in NET-mediated ferroptosis-associated neuronal injury, we manipulated STING activity pharmacologically. Activation of STING using 2'3'-cGAMP restored ferroptotic signaling despite PAD4 deletion, re-suppressing GPX4 expression and re-elevating ACSL4 and 4-HNE levels (Fig. 6K-L). Conversely, STING inhibition with C-176 markedly attenuated ferroptosis markers, mimicking the protective effects observed with PAD4 KO or DNase I treatment (Fig. 6K-L). These results support a critical role for STING activation in promoting NET-derived DNA-associated ferroptotic signaling after TBI.

### Endogenous STING signaling contributes to NET-induced ferroptosis-associated neuronal injury by suppressing the AMPK-Nrf2 antioxidant pathway

**Figure 7:**
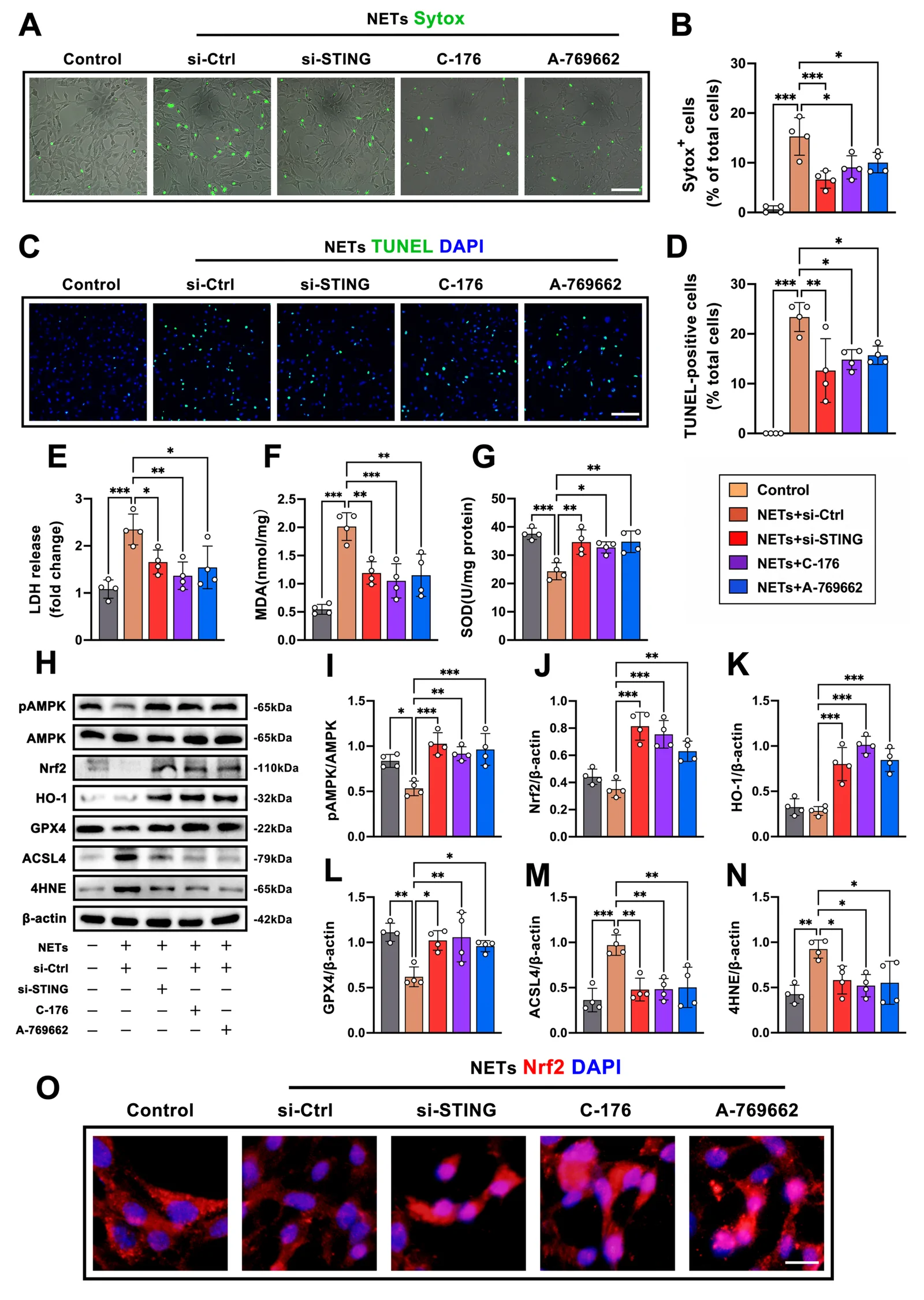
** Endogenous STING signaling mediates NET-induced neuronal ferroptosis via suppression of the AMPK-Nrf2 antioxidant pathway *in vitro*. A, B** Representative images (A) and quantitative analyses (B) of SYTOX Green-positive HT22 cell death following NET stimulation under endogenous STING signaling conditions. HT22 cells were assigned to Control, NETs + si-Ctrl, NETs + si-STING, NETs + C-176, or NETs + A-769662 groups. ***P < 0.001, *P < 0.05, n = 4 per group. Scale bar = 50 μm. **C, D** Representative TUNEL staining images (C) and quantification (D) of TUNEL-positive cells following NET stimulation under the indicated conditions. ***P < 0.001, **P < 0.01, *P < 0.05, n = 4 per group. Scale bar = 50 μm. **E-G** Quantitative analyses of LDH release (E), MDA levels (F), and SOD activity (G) in HT22 cells following NET stimulation with STING knockdown, STING inhibition by C-176, or AMPK activation by A-769662. *P < 0.05, **P < 0.01, ***P < 0.001, n = 4 per group. **H-N** Representative western blot bands (H) and densitometric analyses (I-N) of pAMPK/AMPK, Nrf2, HO-1, GPX4, ACSL4, and 4-HNE expression in HT22 cells subjected to NET stimulation with siRNA-mediated STING knockdown, pharmacological STING inhibition, or AMPK activation. β-actin was used as a loading control. *P < 0.05, **P < 0.01, ***P < 0.001, n = 4 per group. **O** Representative immunofluorescence images showing Nrf2 (red) nuclear localization in HT22 cells following NET stimulation under the indicated conditions. Nuclei were stained with DAPI (blue). Scale bar = 25 μm.

To determine whether endogenous STING signaling contributes to NET-induced ferroptosis-associated neuronal injury, HT22 cells were exposed to NETs under endogenous STING conditions. NET stimulation markedly increased SYTOX-positive and TUNEL-positive cell death in si-Ctrl-transfected HT22 cells (Fig. 7A-D). Genetic knockdown of STING significantly reduced NET-induced cell death, and pharmacological inhibition of STING with C-176 produced a similar protective effect (Fig. 7A-D). In addition, activation of AMPK with A-769662 attenuated NET-induced cell death under endogenous STING conditions (Fig. 7A-D). Consistently, NET exposure increased LDH release and MDA accumulation and decreased SOD activity, whereas si-STING, C-176, and A-769662 each mitigated these oxidative injury readouts (Fig. 7E-G).

We next examined the AMPK-Nrf2 antioxidant axis, a key pathway regulating ferroptosis. NET stimulation suppressed AMPK phosphorylation and reduced Nrf2 and HO-1 expression in HT22 cells, whereas STING knockdown or C-176 treatment restored AMPK-Nrf2-HO-1 signaling under NET exposure (Fig. 7H-K). These changes were accompanied by corresponding alterations in ferroptosis-related markers. NET-treated HT22 cells decreased GPX4 expression and increased ACSL4 and 4-HNE expression, whereas si-STING and C-176 preserved GPX4 and reduced ACSL4/4-HNE accumulation (Fig. 7H, L-N). A-769662 also restored AMPK-Nrf2 signaling and attenuated ferroptosis-associated molecular changes in NET-exposed HT22 cells (Fig. 7H-N). Immunofluorescence analysis further showed that NET exposure reduced Nrf2 nuclear localization, while STING knockdown, C-176, and A-769662 restored Nrf2 accumulation in the nucleus (Fig. 7O). Together, these data demonstrate that endogenous STING signaling contributes to NET-induced ferroptosis-associated neuronal injury and that AMPK activation protects HT22 cells from NET-induced ferroptotic stress under endogenous STING conditions.

### NETs-STING signaling promotes ferroptosis-associated neuronal injury after TBI by suppressing the AMPK-Nrf2 antioxidant pathway

**Figure 8:**
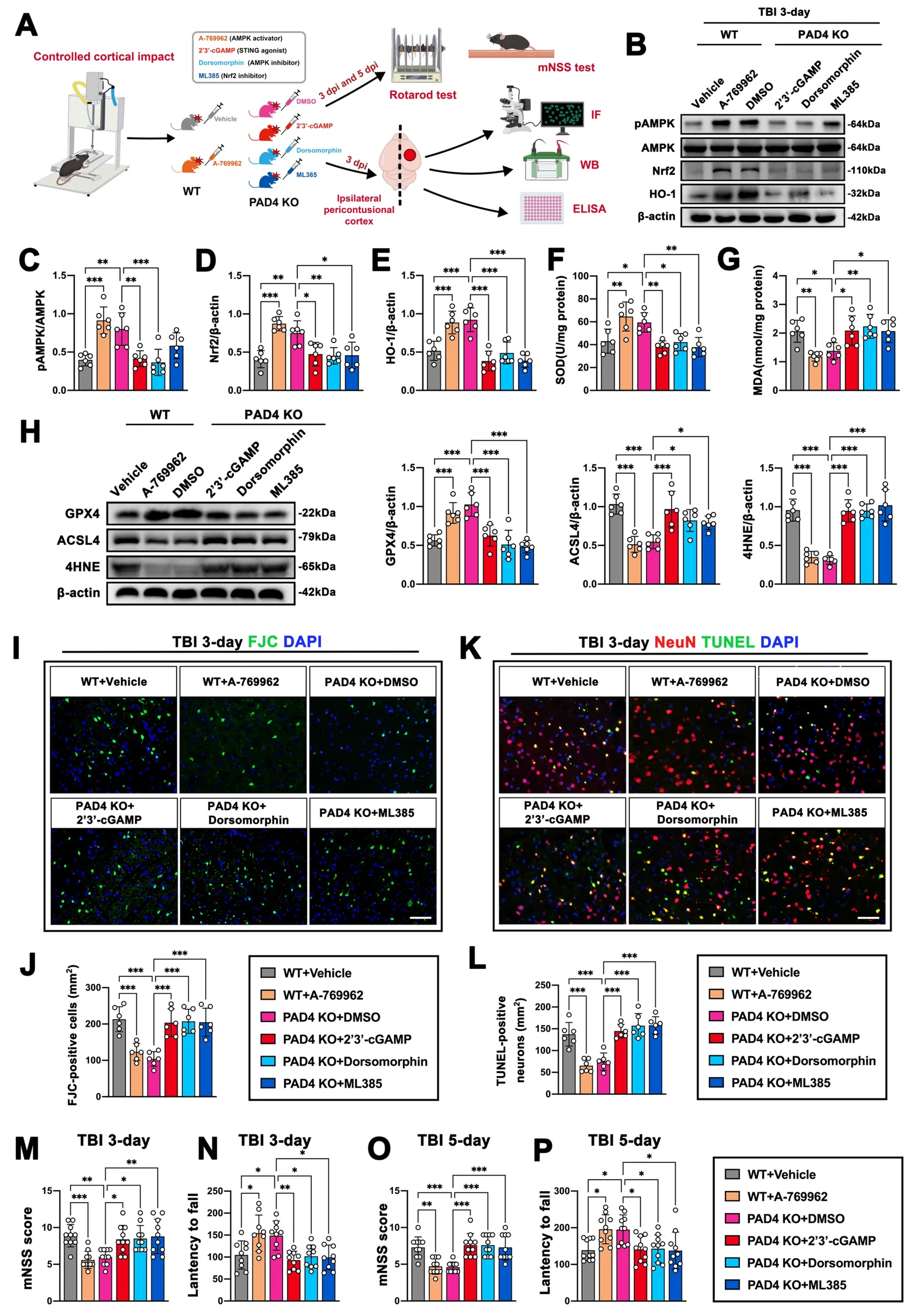
** AMPK-Nrf2 signaling governs STING-mediated ferroptosis-associated neuronal injury and neurological deficits after traumatic brain injury. A** Schematic illustration of the experimental design showing controlled cortical impact, pharmacological modulation of AMPK, STING, and Nrf2 signaling, behavioral assessments, and tissue collection at the indicated time points after TBI. **B-E** Representative western blot bands (B) and densitometric quantification of pAMPK/AMPK (C), Nrf2/β-actin (D), and HO-1/β-actin (E) expression in the ipsilateral peri-contusional cortex from WT and PAD4 KO mice treated with vehicle, A-769662, 2′3′-cGAMP, dorsomorphin, or ML385 at 3 days after TBI. *P < 0.05, **P < 0.01, ***P < 0.001, n = 6 per group. **F, G** Quantitative analyses of SOD activity (F) and MDA levels (G) in cortical tissues at 3 days after TBI. *P < 0.05, **P < 0.01, n = 6 per group. **H** Representative western blot bands and densitometric analyses of GPX4, ACSL4, and 4-HNE expression in the ipsilateral cortex at 3 days after TBI. β-actin was used as a loading control. *P < 0.05, **P < 0.01, ***P < 0.001, n = 6 per group. **I, J** Representative FJC staining images (I) and quantification of FJC-positive cells (J) in the ipsilateral cortex at 3 days after TBI. ***P < 0.001, n = 6 per group. Scale bar = 100 μm. **K, L** Representative images (K) and quantitative analyses (L) of TUNEL-positive neurons co-localized with NeuN in the ipsilateral cortex at 3 days after TBI. ***P < 0.001, n = 6 per group. Scale bar = 100 μm. **M-P** mNSS scores and rotarod latency to fall at 3 days (M, N) and 5 days (O, P) after TBI. *P < 0.05, **P < 0.01, ***P < 0.001, n = 10 per group.

To determine whether AMPK-Nrf2 signaling functionally contributes to STING-mediated ferroptosis-associated neuronal injury *in vivo*, we first evaluated WT and PAD4 KO mice under sham and TBI conditions. In the absence of TBI, PAD4 deficiency did not cause marked baseline changes in AMPK-Nrf2 signaling, ferroptosis-related markers, SOD activity, or MDA levels compared with WT sham mice (Supplementary Fig. S2). After TBI, however, PAD4 KO mice preserved AMPK phosphorylation and Nrf2/HO-1 expression, maintained higher GPX4 and SOD activity, and showed reduced ACSL4, 4-HNE, and MDA levels compared with TBI WT mice (Supplementary Fig. S2). We then manipulated AMPK and STING activity in WT and PAD4 KO mice following TBI (Fig. 8A). Western blot analysis revealed that TBI markedly reduced AMPK phosphorylation and suppressed Nrf2 and HO-1 expression in WT mice, whereas these changes were substantially attenuated in PAD4 KO mice (Fig. 8B-E). Administration of the AMPK agonist A-769662 restored pAMPK, Nrf2, and HO-1 levels in WT animals. In contrast, activation of STING with 2'3'-cGAMP or inhibition of either AMPK (dorsomorphin) or Nrf2 (ML385) eliminated the molecular protection observed in PAD4 KO mice and reduced pAMPK, Nrf2, and HO-1 expression toward WT levels (Fig. 8B-E). Consistent with these changes in upstream signaling, oxidative stress markers displayed parallel patterns. PAD4 KO mice exhibited elevated SOD activity and reduced MDA accumulation compared with WT mice, reflecting diminished oxidative injury (Fig. 8F-G). A-769662 enhanced antioxidant capacity in WT animals, whereas 2'3'-cGAMP, dorsomorphin, or ML385 reversed the oxidative resilience of PAD4 KO mice, reinstating vulnerability to lipid peroxidation. We next assessed ferroptosis-related molecular markers. PAD4 deficiency preserved GPX4 expression and reduced ACSL4 and 4-HNE accumulation after TBI (Fig. 8H). In WT mice, A-769662 significantly increased GPX4 levels and decreased ACSL4 and 4-HNE, confirming a protective effect against ferroptosis-associated damage. Conversely, pharmacological activation of STING or inhibition of AMPK-Nrf2 signaling in PAD4 KO animals markedly decreased GPX4 expression and increased ACSL4 and 4-HNE, effectively reversing the ferroptosis resistance conferred by PAD4 deletion.

Histological analysis further supported these biochemical findings. PAD4 KO mice had markedly fewer FJC-positive degenerating neurons and TUNEL-positive dying neurons in the peri-contusional cortex at 3 days post-injury (Fig. 8I-L). A-769662 produced similar neuroprotection in WT mice. In contrast, 2'3'-cGAMP, dorsomorphin, and ML385 significantly increased neuronal degeneration in PAD4 KO mice, restoring injury levels comparable to WT animals. These cellular effects translated into functional outcomes. PAD4 KO mice showed improved neurological scores and motor performance, and AMPK activation enhanced recovery in WT animals (Fig. 8M-P). Activation of STING or suppression of AMPK-Nrf2 signaling significantly impaired neurological recovery in PAD4 KO mice at both 3 and 5 days post-TBI, further linking this signaling pathway to functional resilience. Collectively, these findings demonstrate that PAD4 deletion protects against TBI-induced ferroptosis-associated neuronal injury primarily by limiting STING activation and preserving AMPK-Nrf2 antioxidant signaling. Forced activation of STING or inhibition of AMPK-Nrf2 is sufficient to abolish this protection, indicating that STING-driven suppression of the AMPK-Nrf2 axis is a key mechanism through which NETs exacerbate ferroptosis and neurological deficits after TBI.

**Figure 9:**
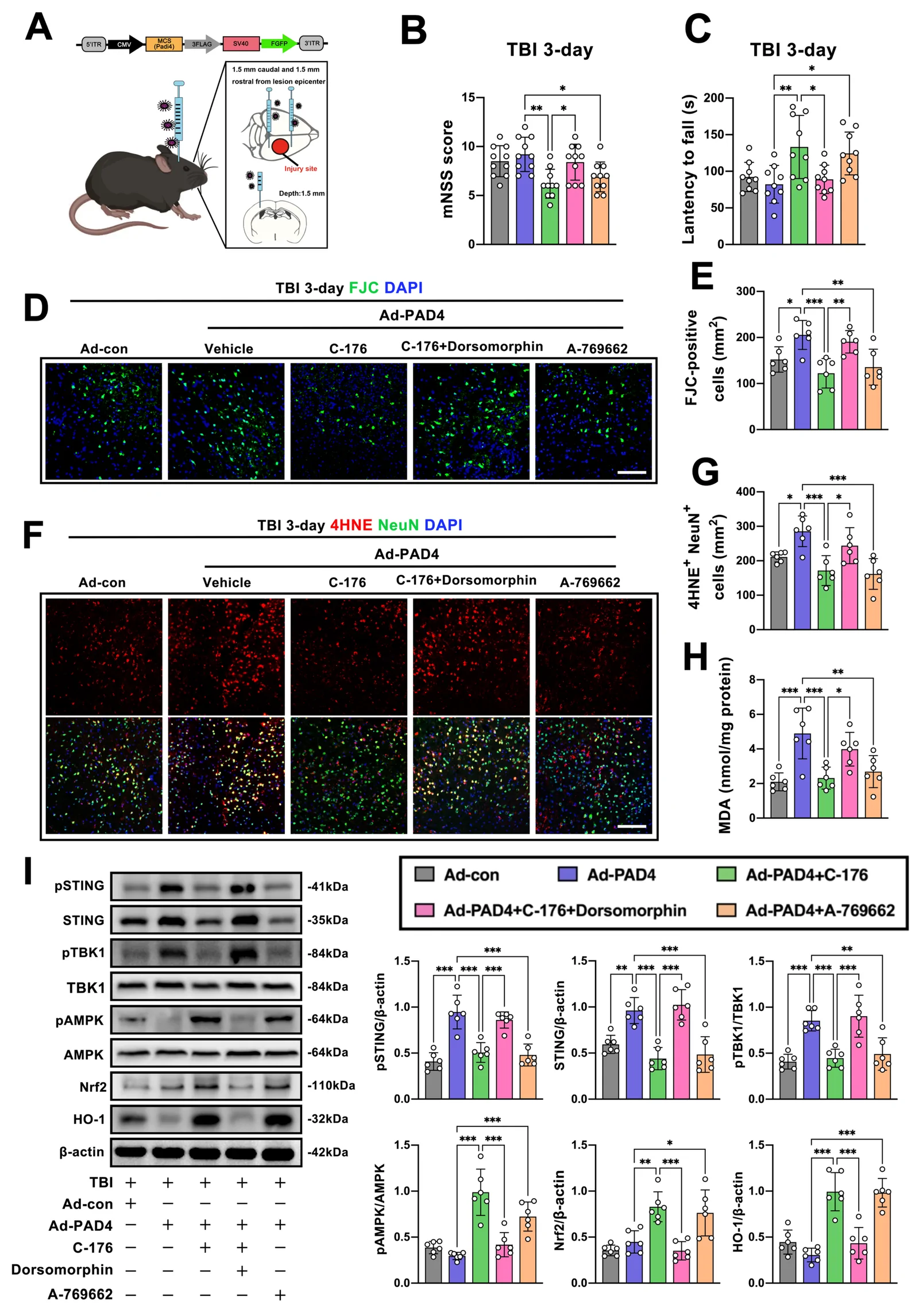
** PAD4 overexpression aggravates ferroptosis-associated neuronal injury through STING-AMPK signaling after traumatic brain injury. A** Schematic illustration of adenovirus-mediated PAD4 overexpression in the ipsilateral cortex and experimental timeline following controlled cortical impact. **B, C** mNSS score (B) and rotarod latency to fall (C) at 3 days after TBI in Ad-con and Ad-PAD4 mice treated with vehicle, C-176, C-176 plus dorsomorphin, or A-769662. *P < 0.05, **P < 0.01, n = 10 per group. **D, E** Representative FJC staining images (D) and quantitative analyses of FJC-positive cells (E) in the ipsilateral cortex at 3 days after TBI under the indicated treatments. Scale bar = 100 μm. *P < 0.05, **P < 0.01, ***P < 0.001, n = 6 per group. **F, G** Representative immunofluorescence images (F) and quantitative analyses (G) of 4-HNE-positive neurons co-localized with NeuN in the ipsilateral cortex at 3 days after TBI. Scale bar = 100 μm. *P < 0.05, ***P < 0.001, n = 6 per group. **H** Quantitative analyses of MDA levels in the ipsilateral cortex at 3 days after TBI. *P < 0.05, **P < 0.01, ***P < 0.001, n = 6 per group. **I** Representative western blot bands and densitometric quantification of pSTING/β-actin, STING/β-actin, pTBK1/TBK1, pAMPK/AMPK, Nrf2/β-actin, and HO-1/β-actin expression in ipsilateral cortical tissues from the indicated groups at 3 days after TBI. *P < 0.05, **P < 0.01, ***P < 0.001, n = 6 per group.

To further test whether PAD4 overexpression enhances NET-related pathology after TBI and to determine whether adenoviral PAD4 expression alone is sufficient to induce NET formation in the uninjured brain, we first compared sham and TBI mice injected with Ad-con or Ad-PAD4. In sham mice, Ad-PAD4 alone did not substantially increase MPO⁺H3cit⁺ NET-like structures, FJC-positive degenerating neurons, MDA accumulation, or neurological deficits compared with Ad-con. After TBI, Ad-PAD4 increased PAD4 and H3cit expression, enhanced MPO⁺H3cit⁺ NET formation, increased FJC-positive neuronal degeneration, and elevated cortical MDA levels compared with TBI + Ad-con mice, whereas mNSS scores and rotarod performance were not further significantly altered (Supplementary Fig. S3). These data indicate that PAD4 overexpression alone is insufficient to establish a NET-enriched pathological state in the uninjured brain, but it amplifies NET formation and ferroptosis-associated neuronal injury in the post-TBI inflammatory environment. We therefore used this TBI-associated PAD4 overexpression model to interrogate downstream STING-AMPK signaling. In Ad-PAD4 mice subjected to TBI, C-176 significantly improved mNSS scores and rotarod latency, whereas co-administration of dorsomorphin partially abolished the behavioral benefit of C-176 (Fig. 9B, C). A-769662 also improved neurological outcomes under PAD4-overexpression conditions (Fig. 9B, C).

Histological and biochemical analyses showed that Ad-PAD4 increased FJC-positive degenerating neurons, 4-HNE⁺NeuN⁺ profiles, and cortical MDA levels after TBI, while C-176 and A-769662 attenuated these ferroptosis-associated injury readouts; dorsomorphin blunted the protective effect of C-176 (Fig. 9D-H). At the molecular level, Ad-PAD4 enhanced pSTING, STING, and pTBK1 expression while suppressing pAMPK, Nrf2, and HO-1. C-176 reduced STING-TBK1 activation and restored AMPK-Nrf2-HO-1 signaling, whereas dorsomorphin weakened the restoration of downstream antioxidant signaling induced by C-176. A-769662 increased pAMPK and reinstated Nrf2/HO-1 signaling despite PAD4 overexpression (Fig. 9I). Together, these findings support that PAD4-enhanced NET formation after TBI aggravates ferroptosis-associated neuronal injury through STING activation and downstream suppression of AMPK-Nrf2 antioxidant signaling.

## Discussion

Here, we identify NET-derived extracellular DNA as a redox-relevant danger signal that promotes neuronal ferroptosis after TBI. Following TBI, massive neutrophil infiltration and NET accumulation occur in both the circulation and injured brain tissue, and by integrating human samples with genetic and pharmacological interventions *in vivo* and mechanistic assays in neurons, we demonstrate that NET-derived DNA activates neuronal STING, which in turn suppresses the AMPK-Nrf2-HO-1 antioxidant program, leading to uncontrolled lipid peroxidation and ferroptosis-associated neuronal injury. Targeting NET formation, STING activation, or AMPK signaling robustly mitigated lipid peroxidation and improved neurological outcomes, demonstrating that neutrophil-derived DNA stress is a key upstream driver of ferroptosis-associated neuronal injury after TBI.

NETs arise from the extrusion of chromatin by activated neutrophils and form web-like extracellular structures^25^. The DNA framework of NETs is decorated with multiple antimicrobial proteins, including histones, neutrophil elastase, MPO and cathepsins, allowing NETs to effectively trap and kill pathogens^47^. NETs have been widely implicated in microvascular dysfunction and sterile inflammation following CNS injury, and accumulating evidence—including our previous studies—has demonstrated that excessive NET formation exacerbates neurological deficits and neuronal loss after traumatic brain injury^5^, ^30^, ^31^, ^33^, ^34^. In particular, pharmacological or genetic inhibition of NET formation was shown to ameliorate neuroinflammation and neuronal death through STING-associated signaling pathways^33^. However, despite these advances, the mechanisms by which NETs directly regulate neuronal vulnerability and death programs remain incompletely understood. In the present study, we found that circulating NET-associated markers were closely correlated with neurological severity and established neuronal injury biomarkers, indicating that systemic NET burden reflects ongoing neuronal injury and loss after TBI. Notably, prominent intracerebral NET deposition was observed in contusional human cortex, where NET-rich regions coincided with neuronal iron accumulation and lipid peroxidation, hallmarks of ferroptosis-associated cell death. While these clinical and histopathological findings indicate a strong association between NETs and neuronal oxidative damage, genetic ablation of PAD4 provided causal evidence that NET formation promotes redox stress and neuronal loss after TBI. By limiting histone citrullination and NET release, PAD4 deficiency markedly reduced oxidative injury and improved neuronal survival, supporting the concept that excessive NET burden functions as an early redox stress amplifier rather than a passive inflammatory byproduct. Collectively, these observations prompted us to explore how NETs translate extracellular inflammatory cues into intracellular redox failure within neurons, with a focus on NET-derived DNA-dependent signaling pathways.

STING is a cytosolic DNA-sensing adaptor localized primarily to the endoplasmic reticulum, where it serves as a central signaling node of the cGAS-STING pathway. Under canonical conditions, STING is activated by cyclic dinucleotides generated in response to cytosolic double-stranded DNA and subsequently translocates to perinuclear compartments, where it engages TBK1 and IRF3 to initiate innate immune signaling and type I interferon responses^48^, ^49^. However, accumulating evidence indicates that STING signaling extends well beyond immune surveillance and may function as a broader cellular stress-response hub^50^, ^51^. Aberrant STING activation has been shown to disrupt mitochondrial homeostasis, promote mitochondrial ROS generation, impair cellular redox balance, and reprogram metabolic stress responses in multiple non-neuronal systems, suggesting that STING may act as an upstream coordinator of redox vulnerability rather than a purely inflammatory mediator^52^, ^53^. In parallel, sustained STING signaling has been linked to alterations in lipid metabolism and lipid peroxidation, raising the possibility that STING shapes susceptibility to ferroptosis-associated injury by lowering intracellular redox thresholds under oxidative stress conditions^54^-^57^.

In sterile injury settings, damage-associated or mislocalized DNA has emerged as a potent trigger of STING activation, positioning STING as a molecular conduit through which extracellular DNA stress is translated into intracellular metabolic and redox dysfunction^58^. Consistent with this framework, our data place neuronal STING downstream of NET-derived extracellular DNA after traumatic brain injury. Genetic suppression of NET formation or enzymatic degradation of extracellular DNA markedly attenuated neuronal pSTING and pTBK1 activation, whereas pharmacological activation of STING was sufficient to reinstate lipid peroxidation and ferroptotic signaling even when NET formation was genetically constrained. Together, these findings extend the functional repertoire of STING beyond its canonical immune role and identify neuronal STING as a redox-ferroptosis amplifier that links NET-derived DNA stress to lipid peroxidation-driven neuronal death.

In the context of ferroptosis, this STING-driven redox vulnerability may be particularly relevant because neuronal ferroptosis is executed when iron-dependent lipid peroxide production overwhelms antioxidant and lipid repair systems. Our data showed that NET-derived DNA-induced STING activation was accompanied by GPX4 depletion, ACSL4 upregulation, increased 4-HNE and MDA accumulation, and elevated Fe²⁺ levels, indicating disruption of the core ferroptotic defense network. These findings support a functional connection between STING activation, pTBK1 induction, AMPK-Nrf2-HO-1 suppression, and ferroptosis-associated neuronal injury, but they do not establish that pTBK1 directly inhibits the AMPK-Nrf2 pathway. Recent studies have shown that STING can promote ferroptosis through multiple mechanisms, including TBK1/NF-κB-dependent ROS generation, TBK1-independent NCOA4-mediated ferritinophagy and iron mobilization, and neutrophil granule molecule-mediated lipid peroxidation in target cells^59^-^61^. Thus, STING may promote ferroptosis-associated neuronal injury not only through innate immune signaling, but also by lowering neuronal resistance to lipid peroxidation and antioxidant failure through both TBK1-dependent and TBK1-independent mechanisms. Future studies using TBK1-specific inhibition or genetic manipulation will be required to determine whether TBK1 directly couples STING activation to AMPK-Nrf2 suppression in neurons after TBI.

Moreover, ferroptosis should be considered as one component of the broader neuronal death landscape after TBI, rather than the sole mechanism of neuronal loss. Other regulated cell death pathways, including apoptosis, necroptosis, and pyroptosis, are also activated during secondary brain injury and may act in parallel or interact with ferroptotic lipid peroxidation. The NET-STING-AMPK pathway may intersect with these death programs through shared inflammatory and redox-sensitive mechanisms. NET-derived DNA-induced STING activation may enhance inflammatory signaling and oxidative stress, thereby creating a permissive environment for pyroptotic, apoptotic, or necroptotic signaling, whereas suppression of AMPK-Nrf2 antioxidant defenses may further amplify mitochondrial dysfunction and lipid oxidative damage. Therefore, the NET-STING-AMPK axis should be viewed as a regulatory hub that may coordinate ferroptosis-associated injury with other regulated cell death pathways, rather than as an isolated linear pathway. In addition, NET-associated molecules beyond extracellular DNA may further shape ferroptotic and inflammatory injury; for example, a recent study showed that NET-associated cathelicidin-related antimicrobial peptide (CRAMP) can form nucleic acid complexes and activate the cGAS/STING pathway in the CNS, supporting the possibility that NET-derived DNA-protein complexes, rather than DNA alone, may contribute to STING-mediated neuroinflammation and ferroptosis-associated neuronal injury^62^.

If STING functions as a redox-ferroptosis amplifier in neurons, a key mechanistic question is how STING signaling disables intrinsic antioxidant defenses that normally constrain lipid peroxidation. Having established that NET-derived DNA activates neuronal STING, we next asked how this activation compromises the neuron's intrinsic antioxidant defense systems, leading us to focus on the AMPK-Nrf2-HO-1 axis as a central regulator of neuronal redox homeostasis. The AMPK-Nrf2-HO-1 pathway is a central antioxidant and metabolic checkpoint that preserves redox homeostasis and limits ferroptosis under metabolic and oxidative stress^63^, ^64^. AMPK acts as a master cellular energy and stress sensor that is activated by ATP depletion, mitochondrial dysfunction, and ROS accumulation—conditions that are particularly prominent in injured neurons^65^. Upon activation, AMPK coordinates adaptive metabolic programs to restore redox balance by suppressing ROS-generating anabolic pathways, promoting mitochondrial quality control, and enhancing antioxidant gene expression, thereby coupling cellular energy status to redox resilience^66^. AMPK activation enhances Nrf2-dependent transcription and reinforces ferroptosis resistance, whereas disruption of this axis sensitizes cells to ferroptotic injury across diverse disease contexts^67^, ^68^. Mechanistically, AMPK facilitates Nrf2 nuclear accumulation and transcriptional activity through phosphorylation-dependent stabilization of Nrf2, inhibition of Keap1-mediated degradation, and modulation of redox-sensitive kinases^63^, ^67^. Through these actions, AMPK amplifies the expression of Nrf2 target genes, including HO-1, SLC7A11, and GPX4, reinforcing glutathione metabolism, iron handling, and lipid peroxide detoxification^11^, ^69^. In neurons, which exhibit high metabolic demand and limited antioxidant reserves, impairment of AMPK-Nrf2 signaling has been closely linked to redox-driven neurodegeneration^70^, ^71^. Building on this framework, our findings indicate that NET-driven STING activation suppresses this protective antioxidant axis after traumatic brain injury. STING activation was associated with reduced AMPK phosphorylation, impaired Nrf2 nuclear accumulation, and diminished HO-1 expression, concomitant with GPX4 depletion, ACSL4 upregulation, and enhanced lipid peroxidation. These coordinated molecular changes suggest that STING activation shifts neurons from an adaptive, AMPK-governed antioxidant state toward a pro-oxidant, ferroptosis-permissive state by dismantling the AMPK-Nrf2-HO-1 checkpoint that normally constrains ROS amplification and lipid peroxide accumulation. Notably, pharmacological activation of AMPK restored Nrf2-HO-1 signaling and markedly attenuated ferroptosis-associated neuronal injury even in the presence of sustained STING activity, underscoring AMPK as a dominant downstream regulatory node capable of overriding STING-induced redox collapse. Together, these data position the AMPK-Nrf2-HO-1 pathway as a critical downstream checkpoint through which STING governs neuronal redox vulnerability after TBI.

Beyond its molecular and cellular effects, the NET-DNA-STING-AMPK axis provides a coherent framework linking ferroptosis-associated neuronal injury to neurological dysfunction after traumatic brain injury. Ferroptosis has been increasingly recognized as a major driver of secondary neuronal loss following CNS injury, with multiple studies demonstrating that iron accumulation, lipid oxidative damage, and GPX4 depletion closely correlate with neuronal degeneration and adverse neurological outcomes^72^-^74^. Experimental inhibition of ferroptosis has been shown to confer neuroprotection and improve functional recovery in diverse models of brain injury, supporting a causal contribution of this death program to post-traumatic neurological decline^75^-^78^. Consistent with these observations, we found that interventions limiting NET formation or attenuating downstream STING signaling were accompanied by reduced ferroptosis-related molecular signatures and neuronal injury, together with significant improvements in sensorimotor and cognitive performance. These findings support the view that NET-driven ferroptotic stress represents a functionally relevant pathological process, rather than a purely histological correlate, in shaping neurological outcomes after TBI.

Despite the comprehensive evidence supporting a central role for NET-derived DNA-STING signaling in regulating neuronal ferroptosis after traumatic brain injury, several limitations should be acknowledged. First, neuronal degeneration after TBI is a multifactorial process involving multiple forms of regulated cell death and injury-related signaling pathways. Although STING inhibition reduced FJC-positive and TUNEL-positive neurons in our study, neuronal injury was not completely restored to sham levels, indicating that STING-related signaling promotes, but does not solely account for, neuronal degeneration after TBI. Thus, the NET-derived DNA-STING pathway should be interpreted as an important contributor to ferroptosis-associated neuronal injury rather than the exclusive mechanism driving neuronal loss in the acute phase of TBI. Second, although our mechanistic experiments focused primarily on neuronal STING signaling, STING is expressed in multiple neural cell types after brain injury. In particular, microglial STING activation may also respond to NET-derived DNA or other NET-associated danger signals and thereby amplify neuroinflammatory responses, cytokine production, and neuron-glia crosstalk. Future studies using cell-type-specific STING manipulation will be needed to distinguish the relative contributions of neuronal and microglial STING signaling to NET-mediated secondary brain injury. Third, although our data establish a causal link between STING activation and suppression of the AMPK-Nrf2-HO-1 antioxidant program using genetic and pharmacological approaches, whether STING modulates AMPK signaling through direct molecular interactions or indirectly via intermediate metabolic or stress-sensing pathways remains unresolved. Fourth, while this study emphasizes extracellular DNA as the principal functional component of NETs, NETs are complex structures containing histones and neutrophil granular proteins, such as MPO and neutrophil elastase (NE), which have been reported to exert cytotoxic and pro-oxidant effects in other contexts. Although our experimental design was intended to isolate the DNA-dependent signaling axis, it does not exclude potential cooperative contributions of other NET constituents under specific conditions. Fifth, although we demonstrate that the NET-STING-AMPK axis influences ferroptosis-related molecular signatures and neurological outcomes during the acute and subacute phases of injury, the spatiotemporal dynamics and cell-type specificity of this pathway across brain regions remain incompletely defined. Future studies employing cell-specific genetic tools, single-cell or spatial transcriptomics, and extended temporal profiling will be essential to clarify how NET-driven immune stress converges on ferroptosis and neuroinflammation to shape neuronal fate. Finally, while DNase treatment, STING inhibition, and AMPK activation represent clinically tractable strategies, further work is required to assess their safety, efficacy, and optimal therapeutic windows across different injury severities.

## Conclusion

In conclusion, TBI triggers robust NET formation in the circulation and injured brain, which exacerbates neuronal ferroptosis and neurological dysfunction. This process is driven by NET-derived extracellular DNA-mediated activation of neuronal STING signaling, leading to impaired antioxidant defenses. Genetic inhibition of NET formation or enzymatic degradation of extracellular DNA alleviated lipid peroxidation and improved neurological outcomes, whereas STING activation aggravated ferroptosis-associated neuronal injury. Notably, pharmacological activation of AMPK effectively counteracted STING-driven ferroptotic stress and preserved neuronal viability. Together, these findings identify NET-derived DNA as a key regulator of neuronal ferroptosis after TBI and highlight neutrophil-driven redox stress as a therapeutic target for secondary brain injury.

## Supplementary Material

Supplementary figures and tables.

## Data Availability

The authors declare that all supporting data are available within the article and the supplemental data or obtained under reasonable requirements.

## Abbreviations

TBI: traumatic brain injury
ROS: reactive oxygen species
NETs: neutrophil extracellular traps
BBB: blood-brain barrier
cGAS: cyclic GMP-AMP synthase
STING: stimulator of interferon genes
CNS: central nervous system
dsDNA: double-stranded DNA
GCS: Glasgow Coma Scale
LPS: lipopolysaccharide
CCI: controlled cortical impact
i.p: intraperitoneal
i.v: intravenous
Ad-PAD4: PAD4 adenovirus
Ad-con: empty adenovirus
mNSS: modified neurological severity score
MWM: Morris water maze
FJC: Fluoro-Jade C
TUNEL: terminal deoxynucleotidyl transferase dUTP nick end labeling
PFA: paraformaldehyde
CTX: cortex
DG: dentate gyrus
PVDF: polyvinylidene difluoride
ELISA: enzyme-linked immunosorbent assay
MDA: malondialdehyde
SOD: superoxide dismutase
DNase I: deoxyribonuclease I
PMA: phorbol 12-myristate 13-acetate
OD: optical density
TBK1: TANK-binding kinase 1
PAD4: peptidylarginine deiminase 4
MPO: myeloperoxidase
NE: neutrophil elastase
PBS: phosphate-buffered saline
Lip-1: Liproxstatin-1
