# Supplementary material for: Neutrophil extracellular traps promote neuronal ferroptosis through STING-mediated AMPK dysregulation after traumatic brain injury
Source: Int J Biol Sci. 2026 Jul 30;22(13):7100–26. doi: 10.7150/ijbs.130882 (PMC13540453; doi:10.7150/ijbs.130882)
Supplement: Supplementary file 1 [file ijbsv22p7100s1.pdf]

Supplementary materials

Part 1: Clinical characteristics of the TBI patient cohort

Table S1. Demographics and clinical characteristics of human brain tissues from acute traumatic brain injury and arteriovenous malformation.

Case	Age	Gender	Cause of Injury	Other injuries	Time Post-Injury(h)	Region of Surgery	GCS
Traumatic brain injury	42	Male	Traffic accident	None	8	Right temporal lobe	5
Traumatic brain injury	55	Male	Traffic accident	None	7	Left frontal lobe	4
Traumatic brain injury	47	Male	Traffic accident	None	10	Left temporal lobe	3
Traumatic brain injury	58	Male	Traffic accident	None	6	Right parietal lobe	5
Traumatic brain injury	31	Male	Traffic accident	None	5	Right temporal lobe	4
Traumatic brain injury	52	Male	Traffic accident	None	7	Right parietal lobe	6
Traumatic brain injury	28	Male	Assault	None	9	Right frontal lobe	7
Traumatic brain injury	31	Male	Assault	None	6	Left temporal lobe	5
Arteriovenous malformation	24	Male	-	None	None	Right parietal lobe	-
Arteriovenous malformation	29	Male	-	None	None	Left parietal lobe	-
Arteriovenous malformation	25	Male	-	None	None	Right temporal lobe	-
Arteriovenous malformation	21	Male	-	None	None	Left parietal lobe	

Part 2: ELISA information

Table S2. ELISA kits used for quantitative analysis in this study

Analyte	Sample type	Manufacturer	Catalog No.	Species
cell-free DNA	Plasma	Invitrogen	P11496	Human/ Mouse
MPO–DNA complexes	Plasma	Roche	11774425001	Human/ Mouse
H3cit	Plasma	Cayman Chemical	501620	Human
UCHL1	Plasma	Elabscience	E-EL-H2377	Human
NEFL	Plasma	Elabscience	E-EL-H6203	Human
Fe^{2+}	Tissue	Elabscience	E-BC-K773-M	Human/ Mouse
MDA	Tissue	Elabscience	E-BC-K025-S	Human/ Mouse
H3cit	Plasma	Cayman Chemical	501620	Mouse
H3cit–DNA complexes	Plasma	Roche	11774425001	Mouse
MDA	Cells	Elabscience	E-BC-K028-M	Mouse
SOD	Tissue / Cells	Cayman Chemical	706002	Mouse
Fe^{2+}	Cells	Elabscience	E-BC-K881-M	Mouse

Part 3: Primary antibody information

Table S3: Primary antibody used for western blot in this study

Antibody	Source	Cat#	Vendor	Country	Dilution	MW (kDa)
Histone H3	Rabbit	9715	CST	USA	1:1000	17
PAD4	Rabbit	ab96758	Abcam	UK	1:1000	74
H3Cit	Rabbit	ab5103	Abcam	UK	1:1000	17
MPO	Rabbit	ab208670	Abcam	UK	1:1000	89
ACSL4	Rabbit	Ab155282	Abcam	UK	1:500	79
GPX4	Rabbit	52455	CST	USA	1:1000	22
4HNE	Rabbit	Ab46545	CST	USA	1:1000	65
pSTING	Rabbit	72971	CST	USA	1:1000	41
STING	Rabbit	13647	CST	USA	1:1000	35
pTBK1	Rabbit	5483	CST	USA	1:1000	84
TBK1	Rabbit	3504	CST	USA	1:1000	84
p-AMPK	Rabbit	ab32382	Abcam	UK	1:1000	65
AMPK	Rabbit	ab32047	Abcam	UK	1:1000	65
Nrf2	Rabbit	ab76026	Abcam	UK	1:1000	110
HO-1	Rabbit	ab52947	Abcam	UK	1:1000	32

Table S4: Primary antibody used for immunofluorescence in this study

Antibody	Source	Cat#	Vendor	Country	Dilution
H3Cit	Rabbit	ab5103	Abcam	UK	1:1000
MPO	Goat	AF3667	R&D Systems	USA	1:500
NeuN	Mouse	ab104224	Abcam	UK	1:1000
4HNE	Rabbit	Ab46545	CST	USA	1:1000
pSTING	Rabbit	72971	CST	USA	1:1000
Nrf2	Rabbit	ab76026	Abcam	UK	1:1000

Part 4: Supplementary figures

Figure S1. Traumatic brain injury induces robust neutrophil recruitment and NET formation in the injured brain and peripheral circulation in mice.

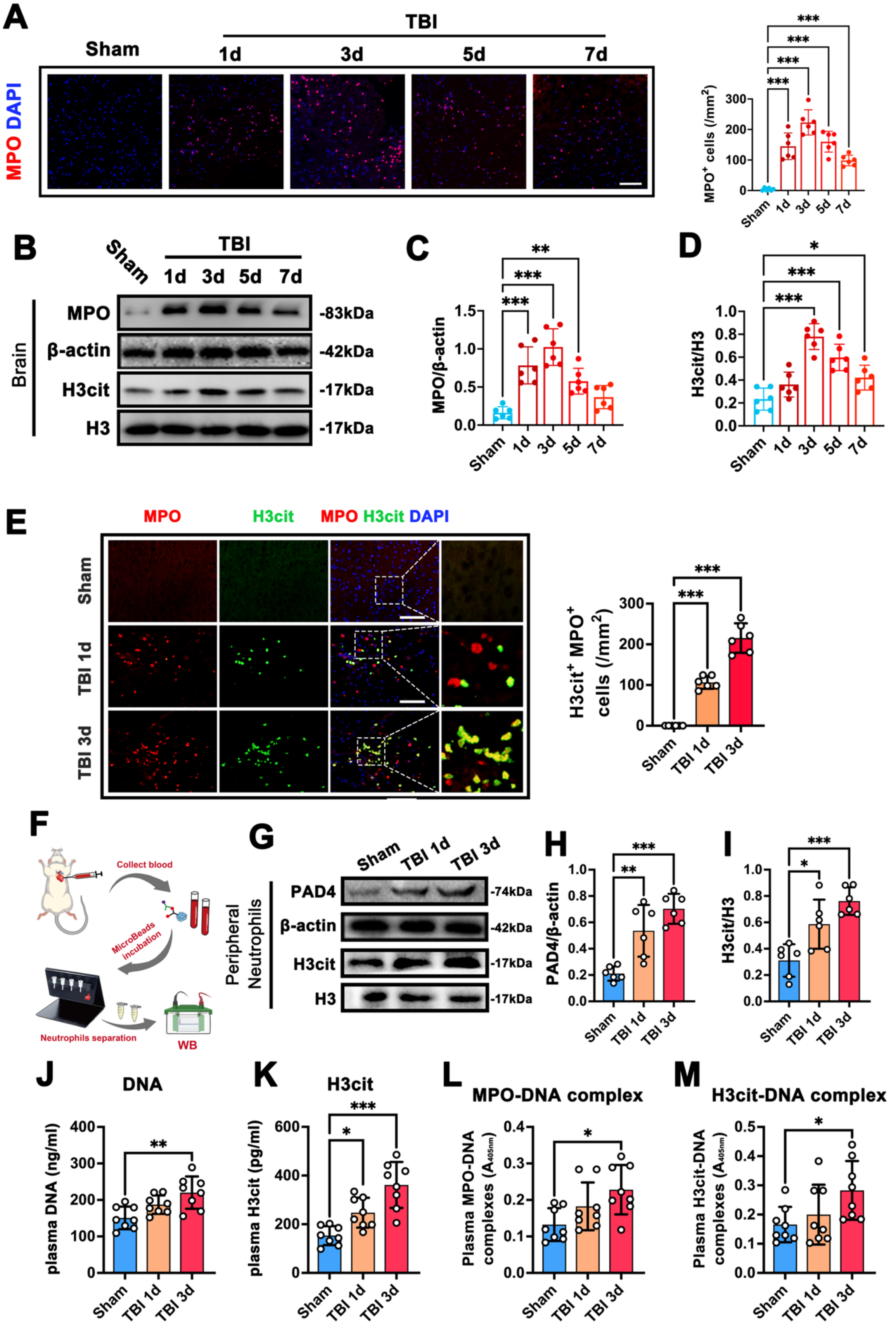

Figure S1. A Representative immunofluorescence images and quantification of myeloperoxidase (MPO)-positive neutrophils in the peri-lesional cortex of sham-operated mice and mice subjected to traumatic brain injury (TBI) at 1, 3, 5, and 7 days post-injury. Nuclei were stained with DAPI. Scale bar = 100 μm. ***$P < 0.001$, $n = 6$ per group. B–D Representative western blot bands (B) and densitometric analyses of MPO/β-actin (C) and citrullinated histone H3 (H3cit)/histone H3 (D) in ipsilateral brain tissues from sham and TBI mice at 1, 3, 5, and 7 days post-injury. β-actin and total histone H3 were used as loading controls. *$P < 0.05$, **$P < 0.01$, ***$P < 0.001$, $n = 6$ per group. E Representative immunofluorescence images and quantification of MPO (red) and H3cit (green) co-positive cells in the peri-lesional cortex of sham and TBI mice at 1 and 3 days post-injury. Nuclei were stained with DAPI (blue). Insets show higher-magnification views of MPO+H3cit+ cells indicative of NET formation. Scale bar = 50 μm. ***$P < 0.001$, $n = 6$ per group. F Schematic illustration of peripheral blood collection, neutrophil isolation, and subsequent western blot analysis. G–I Representative western blot bands (G) and densitometric analyses of PAD4/β-actin (H) and H3cit/histone H3 (I) in peripheral neutrophils isolated from sham and TBI mice at 1 and 3 days post-injury. β-actin and total histone H3 were used as loading controls. *$P < 0.05$, **$P < 0.01$, ***$P < 0.001$, $n = 6$ per group. J–M Plasma levels of cell-free DNA (J), H3cit (K), MPO–DNA complexes (L), and H3cit–DNA complexes (M) measured by ELISA in sham and TBI mice at 1 and 3 days post-injury. *$P < 0.05$, **$P < 0.01$, ***$P < 0.001$, $n = 8$ per group.

Figure S2. PAD4 deficiency preserves AMPK–Nrf2 signaling and attenuates ferroptosis-related oxidative stress after traumatic brain injury.

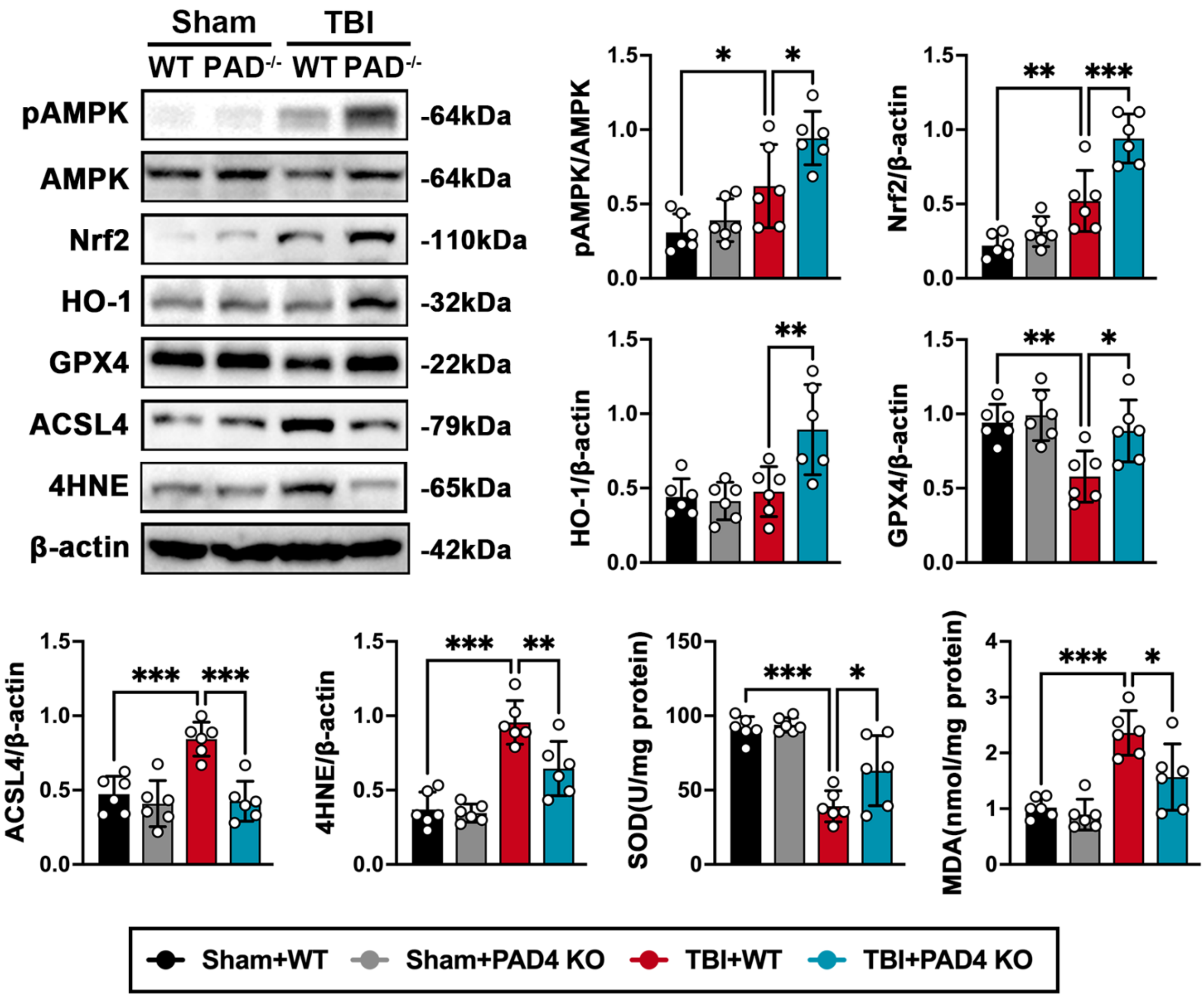

Figure S2. Representative western blot bands and quantitative analyses of pAMPK/AMPK, Nrf2/β-actin, HO-1/β-actin, GPX4/β-actin, ACSL4/β-actin, and 4-HNE/β-actin expression in ipsilateral cortical tissues from Sham + WT, Sham + PAD4 KO, TBI + WT, and TBI + PAD4 KO mice at 3 days after injury. Quantitative analyses of SOD activity and MDA levels in the ipsilateral cortex are also shown. β-actin was used as a loading control. *P < 0.05, **P < 0.01, ***P < 0.001, n = 6 per group.

Figure S3. PAD4 overexpression minimally affects the uninjured brain but exacerbates NET formation and neuronal injury after TBI.

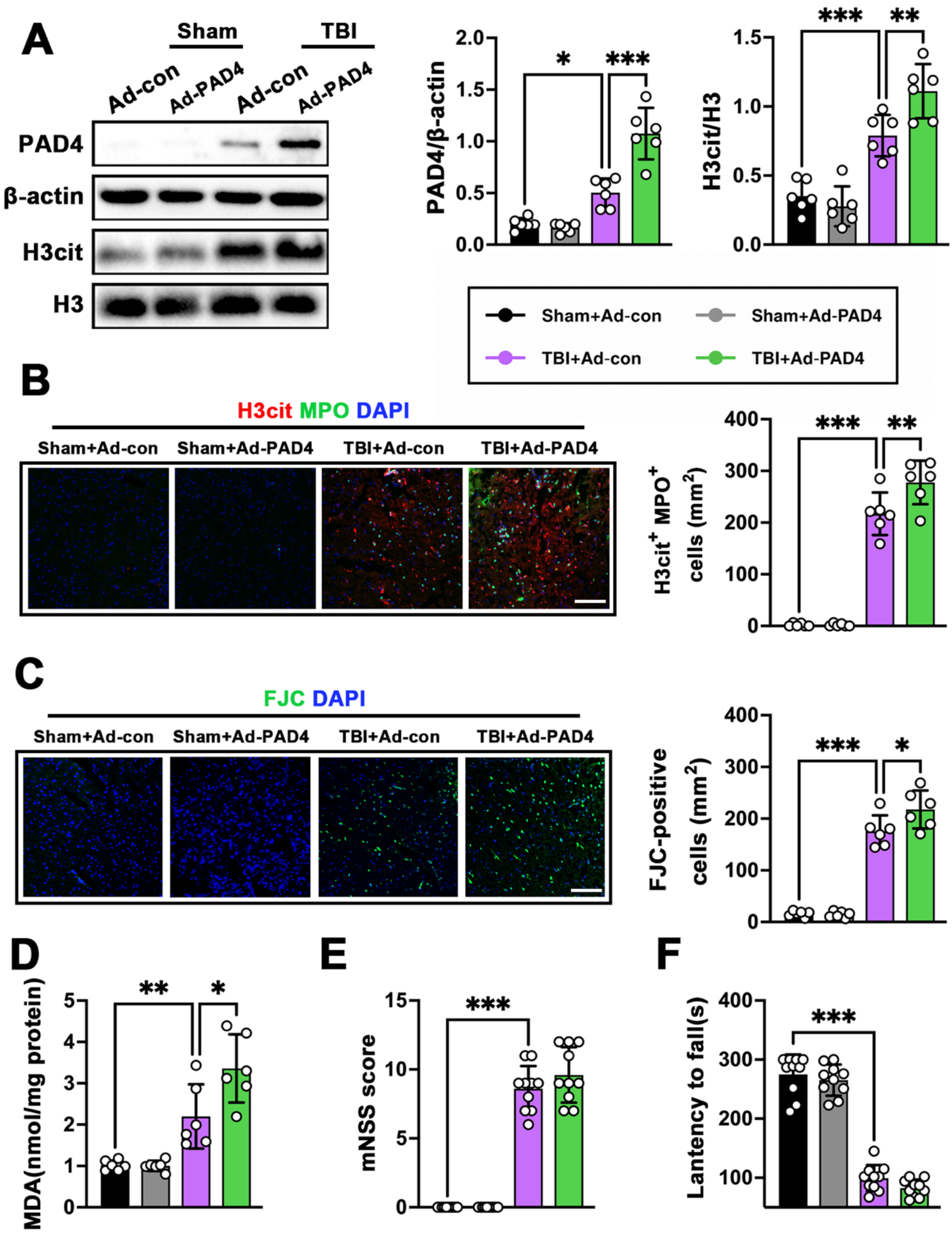

Figure S3. A Representative western blot bands and quantitative analyses of PAD4/β-actin and H3cit/histone H3 in the ipsilateral cortex of Sham + Ad-con, Sham + Ad-PAD4, TBI + Ad-con, and TBI + Ad-

PAD4 mice. β-actin and total histone H3 were used as loading controls. *P < 0.05, **P < 0.01, ***P < 0.001, n = 6 per group. B Representative immunofluorescence images and quantification of H3cit+MPO+ cells in the ipsilateral cortex under the indicated conditions. Nuclei were stained with DAPI. Scale bar = 100 μm. **P < 0.01, ***P < 0.001, n = 6 per group. C Representative FJC staining images and quantification of FJC-positive cells in the ipsilateral cortex under the indicated conditions. Nuclei were stained with DAPI. Scale bar = 100 μm. *P < 0.05, ***P < 0.001, n = 6 per group. D Quantification of MDA levels in the ipsilateral cortex. *P < 0.05, **P < 0.01, n = 6 per group. E, F mNSS scores (E) and rotarod latency to fall (F) under the indicated conditions. ***P < 0.001, n = 10 per group.
